# Live exotic animals legally and illegally imported via the main Dutch airport and considerations for public health

**DOI:** 10.1371/journal.pone.0220122

**Published:** 2019-07-24

**Authors:** Annika van Roon, Miriam Maas, Daniela Toale, Nedzib Tafro, Joke van der Giessen

**Affiliations:** 1 Centre for Infectious Disease Control, National Institute for Public Health and the Environment (RIVM), Bilthoven, the Netherlands; 2 Border inspection, Netherlands Food and Consumer Product Safety Authority (NVWA), Amsterdam, The Netherlands; The Scripps Research Institute, UNITED STATES

## Abstract

The trade in live animals and animal products is considered one of the major drivers of zoonotic disease emergence. Schiphol airport in the Netherlands is one of the largest European airports and is considered a main hub for legal and illegal import of exotic animals. However, so far there is little information about what pathogens these imported animals might carry with them. Therefore, this study aimed to assess the zoonotic risks of exotic animals imported into the Netherlands through Schiphol airport in 2013 and 2014. Based on a previous list of highly prioritised emerging zoonoses for the Netherlands (EmZoo list), WAHID and Promed databases, literature and expert opinions, a list of 143 potentially relevant zoonotic pathogens was compiled. In a step-wise selection process eighteen pathogen-host combinations that may pose a public health risk by the import of exotic animals via Schiphol airport were identified and these were assessed by expert elicitation. The five pathogens with the highest combined scores were *Salmonella* spp., Crimean-Congo haemorrhagic fever virus, West Nile virus, *Yersinia pestis* and arenaviruses, but overall, the public health risk of the introduction of these exotic pathogens into the Netherlands via the legal import of exotic animals was considered low. However, the vast majority of imported exotic animals were imported by trade companies, increasing the risk for specific groups such as retail and hobbyists/pet owners. It is expected that the risk of introduction of exotic zoonotic pathogens via illegal import is substantial due to the unknown health status. Due to changing trade patterns combined with changing epidemiological situation in the world and changing epidemiological features of pathogens, this risk assessment needs regular updating. The results could give directions for further adjusting of health requirements and risk based additional testing of imported exotic animals.

## Introduction

Trade of exotic animals has existed since prehistory [1]. Due to the tremendous expansion of the human population in the last decades, economic growth and globalization, the demand for exotic animals increased and likewise the scale and extent of the exotic animal trade enlarged [2],[3]. The trade in domestic or exotic animals (products) has been indicated as one of the major drivers of zoonotic disease emergence [4],[5].These zoonotic pathogens have represented 75% of all emerging infectious diseases in the past decade [6]. The majority (60%) of these emerging infectious diseases are due to pathogens with wildlife origins [7], [8]. A similar percentage was obtained, when emerging zoonotic pathogens relevant for the Netherlands were ranked using multicriteria decision analyses: of the 86 pathogens ranked, more than 60% originated from wildlife [9]. Many zoonotic pathogens are present in third countries (i.e. non-European Union member states), and the international trade of exotic animals is one of the risk factors to introduce these pathogens [5]. There have been various examples in a wide range of species where the legal and illegal trade in live exotic animals raised public health concerns [10], [11], [12], [13]. Most of the trade in exotic animals is legal, but a significant part is not [14]. It is thought that the global illegal wildlife trade is worth at least 19 billion US dollars per year, which makes it the fourth largest illegal business after narcotics, counterfeiting and human trafficking [15],[16].

For import of live animals into the EU, health certificates or trade documents, issued by the competent Veterinary Authority of the exporting country, are required. The requirements are related to the country of origin and animal species. For example the international legal trade in many exotic animals, especially in mammals and birds, is only possible between registered premises as zoological gardens and bird parks. Regulations on the trade in endangered wild animals are defined in the EU Wildlife Trade Regulations [17] and the Convention on International Trade in Endangered Species of wild flora and fauna (CITES) [18].

Few risk assessments have been performed regarding animal imports and even fewer focus on exotic animal imports and the zoonotic health risks to humans. All studies emphasized the need for more research as the lack of data hampers risk assessment studies [2], [19], [20]. To better understand the risk of the global movement of exotic animals, knowledge is required about species of animals imported, volumes moved and trade routes [21].

Schiphol airport in the Netherlands is one of the largest European airports and is considered one of the main hubs for both legal and illegal import of exotic animals [22]. However, there are no studies published with numbers and species of exotic animals imported into the Netherlands and what pathogens these animals might carry. Therefore, in a collaborative project, an inventory was made of the numbers and species of exotic animals legally imported into the Netherlands in 2013 and 2014 at Schiphol airport, and the risk of introduction of pathogens relevant for livestock and public health were assessed. The inventory and the risk for livestock are described elsewhere [23]. In the current study, we aimed to assess the zoonotic risks of live exotic animals, defined as non-native animals that were not fish or invertebrate classes of animals, imported into the Netherlands. This was done by ranking animal host-pathogen combinations based on literature and expert opinion. Furthermore, an inventory was made of the numbers and species of exotic animals seized at Schiphol airport between January 2012 and December 2014 with the aim to assess the illegal import of exotic animals via Schiphol airport and to identify differences between legal and illegal trade.

## Material and methods

### Hazard identification

#### Import of exotic animals into the Netherlands

Numbers and animal species of exotic animals legally imported into the Netherlands between 2013–2014 were previously reported [23]. Briefly, the database contained 1404 consignments (i.e. shipment of animals subdivided according to species), representing a total number of 490,750 exotic animals. A little less than half of these animals (43%) was destined for the Netherlands, a small number (4%) was destined for other EU countries and the rest (53%) was in transit to third countries. The majority of the animals imported in the Netherlands were reptiles (93.8%), followed by amphibians (5.8%), birds (0.06%) and mammals (0.4%). The animals originated from 25 different countries, with most animals from the USA (78.8%), Vietnam (5.1%), Indonesia (3.5%) and Tanzania (3.1%).

#### Zoonotic pathogens of concern in exotic animals legally imported into the Netherlands

**Fig 1:**
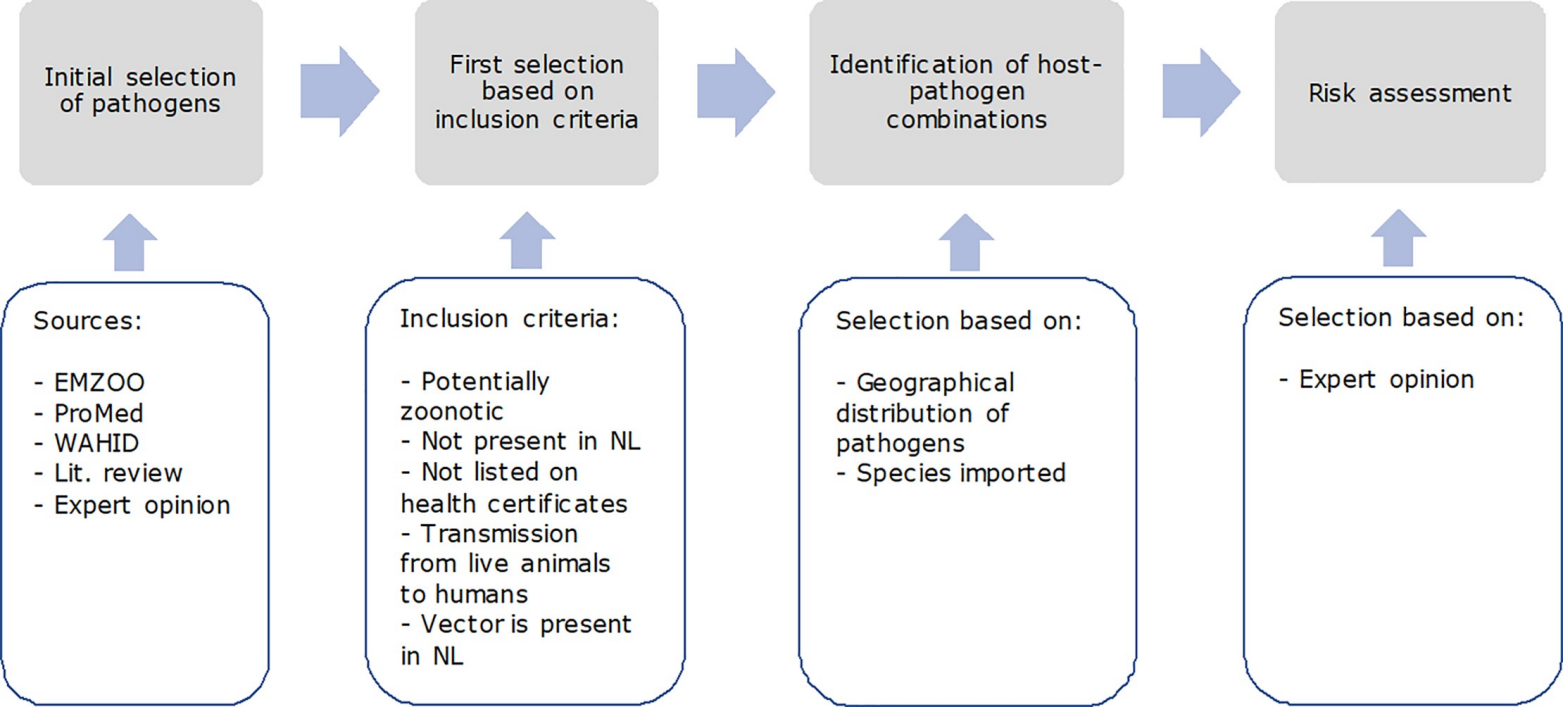
Stepwise selection of zoonotic pathogens of concern in exotic animals legally imported into the Netherlands.

Pathogen-host combinations that may pose a public health risk by the import of exotic animals were identified following a stepwise selection process (Fig 1).

1 Initial selection of pathogens

To start the risk assessment, a longlist was compiled of pathogens that may pose a public health risk associated with the import of exotic animals. Multiple sources were used to compile this longlist. First, 86 zoonotic pathogens were included that had previously been selected and ranked as potential threats to public health in the Netherlands, hereafter referred to as "the EmZoo list" [9]. In addition, for the ten countries that exported the largest total number of animals to the Netherlands, a search was done on ProMed-mail and the WAHID database, using search terms “[country name] AND zoonosis”. The list was supplemented with pathogens from literature studies on pathogens in exotic animals and by expert opinion.

2 First selection based on inclusion criteria

As a second step, pathogens on the longlist were included in the risk assessment when these pathogens complied to the following inclusion criteria:

- Zoonotic potential of the pathogen is proven (i.e. human cases have been described).
- Pathogen is absent in the Netherlands OR is present but more virulent genotypes do exist in other parts of the world.
- Pathogen is not listed on the health certificates of the imported animals and thus not already tested for/controlled.
- The pathogen can be directly transmitted from live animals to humans or via a vector or intermediate host
- The vector or intermediate host is present in the Netherlands for those pathogens that need a vector or intermediate host for transmission.

3 Identification of host-pathogen combinations

This step consisted of two parts: a check whether the pathogens were present in the list of countries that exported to the Netherlands, and a check whether the animal species that were imported in the Netherlands are susceptible to the pathogens. Pathogens were included in the risk assessment when, based on literature, they are present in a country that exported animals to the Netherlands In 2013 or 2014, and when the imported animals are susceptible to infection with these pathogens.

4 Risk assessment

Information was collected about the transmission route of the pathogen via animals to humans, the numbers of relevant animal species that were imported from the exporting countries, whether animals were imported into the Netherlands or in transit, and the disease the pathogen causes in humans. Then the pathogens were assessed by expert elicitation at the National Institute for Public Health and the Environment (RIVM). Experts of various disciplines including virology, vector-borne zoonoses, parasitology and wildlife-born zoonoses were asked to score the public health risk of each pathogen relative to the other pathogens. A score of 1 represents the lowest public health risk among all pathogens considered and 4 the highest public health risk among all pathogens considered. The involved experts were asked to give one score ranging from 1 to 4 for each pathogen while taking into account: the probability of introduction of the infectious pathogen, the probability of transmission of the pathogen to humans in the Netherlands and the impact of human disease. The scores given by the different experts are presented as a range per pathogen e.g. a pathogen receives score 2 from two experts and score 3 from the other two experts, leading to a final score of 2–3 for this pathogen. One expert scored only nine of the 18 pathogens as the other pathogens were outside her field of expertise.

### Illegal import of live animals into the Netherlands

Reports about seizures of live illegally imported animals at Schiphol airport between January 2012 and December 2014 were received from the Netherlands Food and Consumer Product Safety Authority (NVWA). These reports consisted of e-mail correspondence between NVWA staff members and customs about seizures of illegally imported animals or animal products, photographs of the seized animals and receipts for health checks and quarantine required for the animals. Only seizure reports that met the following criteria were included: (1) the seizure concerned live non-native animals that were not fish or invertebrate classes of animals, (2) there was no evidence that the animals were accidentally imported, e.g. a bat free-roaming in an airplane, (3) animals were seized because they were illegally imported and not because of welfare issues. The reports were documented in a database including animal species, country of origin, results of diagnostic tests that were performed, transport method and presence/absence of health certificates. Descriptive analysis was performed using Microsoft Excel 2010.

## Results

### Zoonotic pathogens of concern in exotic animals legally imported into the Netherlands

**Fig 2:**
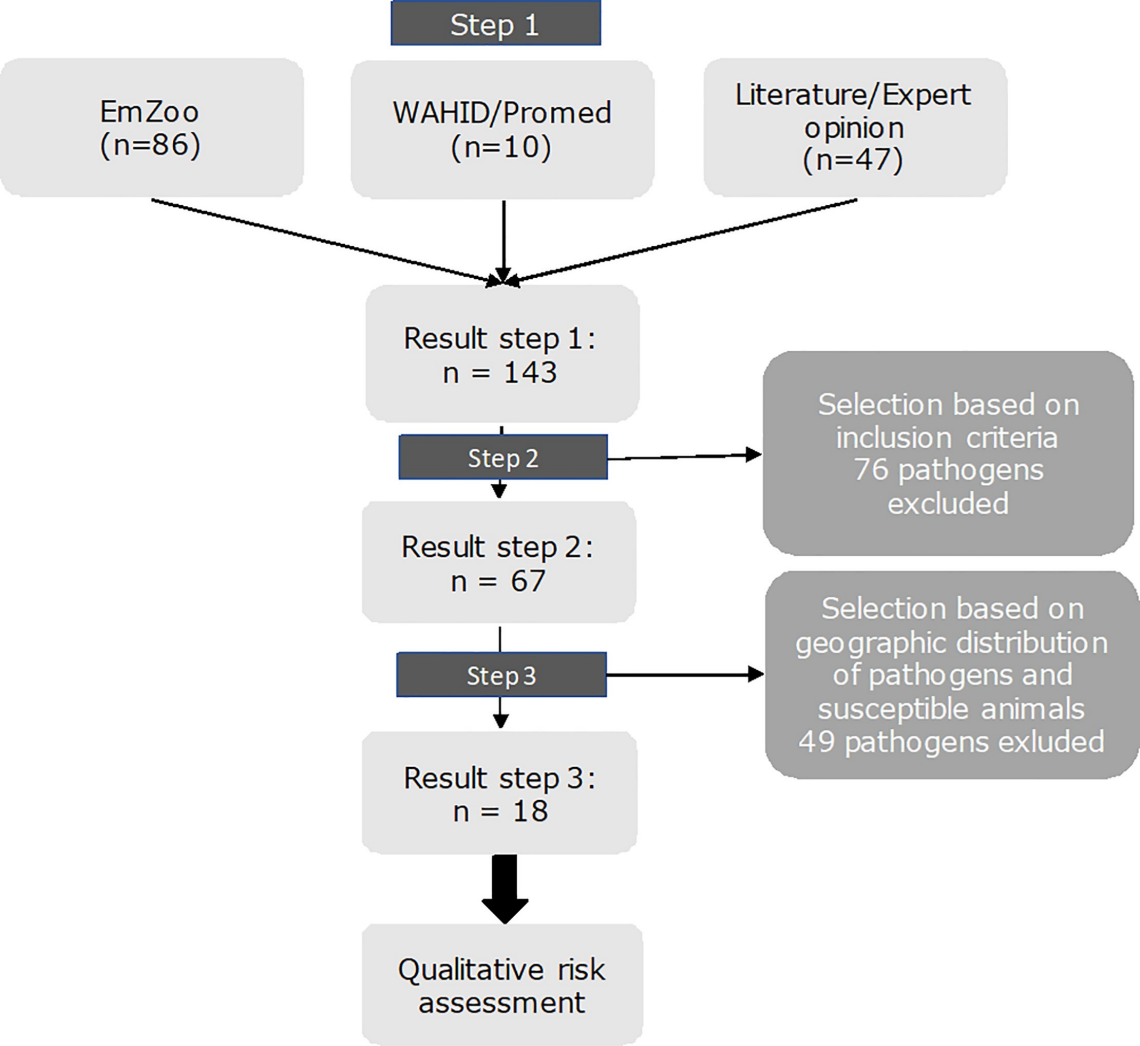
Flow chart presenting the selection steps of potentially relevant pathogens.

Based on the first step as described above, a list of 143 potentially relevant zoonotic pathogens was compiled. In the subsequent selection steps S1, S2 and S3 Tables, this list was reduced to a final list of 18 pathogens that are considered potentially relevant pathogens for public health of the legal import of exotic animals into the Netherlands (Fig 2).

### Risk assessment

The remaining 18 pathogens were scored relative to each other by expert elicitation. The information that was used for scoring the pathogens, in addition to the expert elicitation, is presented in S3 Table. The range of the expert opinions of each pathogen was determined and subsequently the pathogens were ranked according to this score Table 1. In case of equal concluding scores, the pathogens were ranked in alphabetical order. None of the pathogens were scored at the highest level, and the five pathogens with the highest combined scores were *Salmonella* spp., Crimean-Congo haemorrhagic fever virus, West Nile virus, *Yersinia pestis* and arenaviruses.

**Table 1:** Eighteen potentially relevant pathogens scored relative to each other and ranked by expert opinion.

Pathogens	Animal classes^a^	Country of origin^b^	Range^c^	
			Min	Max
*Salmonella* spp. ^d^	Birds, reptiles, amphibians	Worldwide	2	4
Crimean-Congo haemorrhagic fever virus	Birds, mammals, reptiles	China, Egypt, Kenya, South Africa, Tanzania, UAE	2	3
West Nile virus	Birds	Brazil, Canada, South Africa, Tanzania, USA	2	3
*Yersinia pestis*^*e*^	Mammals	Argentina, Egypt	2	3
Arenaviruses^e^	Mammals	Argentina	1	3
Eastern equine encephalitis virus	Birds, reptiles	Brazil, Canada, Columbia, Ecuador, USA	2	2
Japanese encephalitis virus	Birds	Philippines, Singapore	2	2
Western equine encephalitis virus	Birds, reptiles	Argentina, Canada, Guyana, Peru, Suriname, USA	2	2
Rocio virus	Birds	Brazil	1	2
Ross river virus	Mammals	Indonesia	1	2
Saint Louis encephalitis virus	Birds	Argentina, Brazil, Canada, Cuba, Peru, Suriname, USA	1	2
Sindbis virus^e^	Birds	Tanzania	1	2
Venezuelan equine encephalitis virus^e^	Birds	Peru, USA	1	2
*Echinococcus granulosus*^*e*^	Mammals	Chile	1	2
*Rickettsia africae*/ African tick bite fever^e^	Mammals	South Africa	1	1
*Rickettsia typhii*^*e*^	Mammals	Mexico	1	1
*Rickettsia rickettsii*^*e*^	Mammals	Argentina	1	1
T-cell lymphotropic virus 1/HTLV-1	Mammals	Ghana, Peru, Tanzania, USA	1	1

^a^ Animal classes that were imported into the Netherlands in 2013–2014 that could play a role in the transmission of the pathogen.

^b^ Countries where pathogen is present and where animals were imported from into the Netherlands in 2013–2014

^c^ A scale of 1 to 4 was used, where 1 represents the lowest risk and 4 the highest risk

^d^ Only scored by two of the experts as the pathogen was outside the field of expertise of one expert and the other expert indicated that there was not enough information to decide on the risk

^e^ Only scored by three of the experts as the pathogen was outside the field of expertise of one expert

### Illegal import of exotic animals into the Netherlands 2012–2014

Two hundred forty one seizures of animals or animal products that were imported or in transit at Schiphol airport were identified as illegal. These seizure reports contained information about numbers of animals, species of animals, country of origin, results of diagnostic tests, transportation method, the presence or absence of health certificates or (wrong) import papers, whether it concerned CITES listed species and destination of animals after seizure. Not all seizure reports contained all information. Of the 241 seizures, only 38 met the inclusion criteria and were considered illegally imported live animals that were non-domesticated and not fish or invertebrate classes of animals Table 2. The 38 seizure reports consisted of 58 consignments and 514 individual animals.

**Table 2:** Number of consignments (C) and individual animals (I) per class and country of origin that were attempted to be illegally imported into the Netherlands or were in transit through Schiphol airport between the years 2012–2014.

	Amphibia C (I)	Aves C (I)	Reptilia C (I)	Total C (I)
Canada		1 (3)		1 (3)
China			5 (16)	5 (16)
Curacao		1 (5)	2 (6)	3 (11)
Guyana			2 (203)	2 (203)
Indonesia		3 (22)		3 (22)
Iran		1 (2)		1 (2)
Iraq		1 (2)		1 (2)
Mexico		5 (76)		5 (76)
Morocco		3 (8)		3 (8)
Netherlands Antilles			1 (7)	1 (7)
Panama			1 (1)	1 (1)
Suriname		24 (127)		24 (127)
Tunisia			1 (1)	1 (1)
Turkey		2 (15)		2 (15)
USA	4 (19)			4 (19)
Unknown			1 (1)	1 (1)
Total	4 (19)	41 (260)	13 (235)	58 (514)

C: Number of consignments (i.e. shipment of animals subdivided according to species)

I: Number of individual animals

Most consignments of the illegal import originated from Suriname (41%), all concerning birds, followed by five consignments of reptiles from China (9%) and five consignments of birds from Mexico (9%). When analysing the number of individual animals, the top three of countries for illegally imported animals are Guyana, Suriname and Mexico. In contrast to these illegal imports, in the same period, most consignments of legally imported animals originated from Indonesia, Suriname and the USA. On the individual animal level, the top three countries of origin for legally imported animals were Peru, the USA and Vietnam [23].

The reported animals were exotic amphibians, birds and reptiles. Of the 58 consignments, 11 consignments (19%) concerned CITES-listed species. The majority of the consignments concerned birds (71%), followed by reptiles (22%) and amphibians (7%). In contrast to legal import, in the same period, 1404 consignments of exotic animals with in total 490,750 individual animals were legally imported into the Netherlands or in transit to other countries. The consignments of legally imported animals concerned reptiles (65%), amphibians (18%), birds (11%) and mammals (5%) [23].

According to European legislation, all birds that are confiscated should be tested for Avian Influenza and Newcastle Disease. Ninety-five percent of the confiscated birds tested negative for Avian Influenza and of the remaining 5% the results were not documented. For confiscated reptiles and amphibians, there are no health requirements.

In this project, a rough estimate of the number of illegally imported exotic animals was made based on the expert opinion of an expert on wildlife crime and the expert opinion of an expert ranking member at EcoHealth Alliance. The first opinion was that the entire illegal trade can be extrapolated from the seizures, the number of seized animals is 10% of the entire illegal trade. The second opinion was that illegal trade is equivalent to the legal trade. This gives a broad estimate of 1,700 to 245,000 illegally imported and transited animals per year between 2013–2014.

## Discussion

This study aimed to assess the zoonotic risks of exotic animals imported into the Netherlands via Schiphol airport by literature and expert elicitation. Ideally, the risk factors for introduction and transmission of zoonotic pathogens would be systematically weighted and compared, but there were no data available to apply this method. The five pathogens that scored highest were: *Salmonella* spp., Crimean-Congo haemorrhagic fever virus, West Nile virus, *Yersinia pestis* and arenaviruses. However, the overall public health risk of these pathogens via the legal import of exotic animals was considered low reflected by the low scores in general.

*Salmonella* spp. was scored highest, although scoring *Salmonella* spp. was difficult, as over 2500 different Salmonella serotypes exist, which differ in virulence [24]. While some serotypes are host-specific, all mammals, birds, reptiles and amphibians can become infected with *Salmonella* spp. A large number of reptiles (468,621) as well as birds (2,938) and amphibians (17,292) were imported into (43%) or transited through (57%) the Netherlands in 2013–2014. Since exotic animals are not being tested for *Salmonella* spp. and a large number of animals are imported by trade companies (99.8%) [23] and mostly destined for the pet industry, the probability of exposure of humans to (rare and exotic) *Salmonella* spp. is high. Salmonellosis has been linked to pet turtles in the USA [25], to bearded dragons in Canada [26] and pet reptiles in Europe [27]. In Sweden, a rise in the number of reptile-associated *Salmonella* cases was observed since 1996 when the import regulations no longer required certificates stating that imported reptiles were free of *Salmonella* [28]. In the Netherlands, reptile-associated salmonellosis was studied during a 30-year period (1985–2014) and a significant annual increase of 19% was found in reptile-associated salmonellosis in humans [29]. Amphibians are also associated as a source of human *Salmonella* [30], but in our study amphibians were not assumed to be as relevant as reptiles as much lower numbers were imported. However, this number might be highly underestimated as many amphibians are imported along with, and classified as, live ornamental fish [23]. Additional risk is that many amphibians and also reptiles are often caught in the wild and exported. In order to assess the risk of amphibians properly, the reporting of imported amphibians should be improved.

Scoring CCHFV was complicated as it is still uncertain if the imported exotic animal species, although they are a potential reservoir, develop a viremia high enough for transmission of CCHFV and, thus, can play a role in the transmission of CCHFV. Therefore, predominantly the risk of transporting virus-infected larvae and nymphs when importing exotic animals should be considered. For an ongoing transmission concerning multiple persons, both competent hosts and a vector population are needed. The *Hyalomma* tick, the main vector of CCHFV, cannot (yet) establish in the Netherlands due to the cold and wet climate, but CCHFV has also been found in other tick species, including *Rhipicephalus sanguineus* [31],[32], which is incidentally found in the Netherlands. There were birds (403), testudines (1092) and rodents (217) imported or transited through the Netherlands in 2013–2014 that came from countries where CCHFV is present. A small part (39) of these testudines concerned *Testudo graeca* [23], which have been found before to carry *Hyalomma aegyptium* ticks with a high prevalence (30.2%) of CCHFV in Turkey and Syria [33]. As no animal health certificates are required for reptiles and there are no regulations about tick control, there is a risk of importing CCHFV into the Netherlands with infected ticks and potentially with viraemic exotic animals. Scores were also relatively high, because the human impact after exposure is high, although more information is needed to better assess the risk.

The risk of West Nile Virus (WNV) from imported exotic animals for public health was scored second highest together with CCHFV and *Yersinia pestis*. WNV can either be introduced into the Netherlands by the import of an infectious vector or by the import of an infected bird with a viremia high enough to infect endemic mosquitoes, since the competent vectors are present in the Netherlands. The probability that WNV will be introduced in the Netherlands by the import of an infectious vector is very low as consignments of live animals from third countries have to be sprayed with insecticidal aerosols against vectors [34]. When it concerns non-detected illegal import these animals are not sprayed. But even in the latter case, the chance of import of mosquitoes is considered low due to the fact that mosquitoes do not stay on the host after blood feeding. Nevertheless, exotic mosquitos are regularly found at the EU airports. In case an infectious vector is imported, it must be able to survive (long enough) in the Netherlands in order to further transmit WNV. The probability that WNV will be introduced in the Netherlands by the import of an infected bird with a viremia high enough to infect Dutch mosquitoes is also not very high as most birds are only viraemic for a few days [35]. However, in the Netherlands there is a range of suitable vectors present and there are still questions on the role of mammals, reptiles and amphibians in the lifecycle of WNV, complicating a risk assessment of this pathogen. Therefore, also keeping in mind the rapid spread of WNV reported in other countries, WNV is one of the top five pathogens on the priority list. When humans become infected with WNV, disease can be severe, although most infections are asymptomatic.

*Yersinia pestis* can be introduced in the Netherlands by infected rodents and a wide range of flea species. More than 200 species of rodents and lagomorphs have been implicated as reservoirs for *Yersinia pestis*, but the exact number of rodent species that are more than accidental reservoirs is uncertain [36]. As fleas, that are the main transmission route, are permanent ectoparasites, which usually stay on their host, the import of fleas with rodents is to be expected. Human plague is a very serious illness that can lead to death, but can be treated with commonly available antibiotics [37]. In 2013–2014, 226 rodents from Egypt and Argentina were imported into (217), or transited through (9), the Netherlands. In the USA, Egypt and Argentina the presence of sylvatic foci of the plague is suspected [38],[39]. On the general veterinary health certificate that accompanied these legally imported animals, it is stated that animals should be either born in captivity or have been held in captivity for at least six months. Thus, there is a chance that these animals were sourced from the wild, though this is not very likely.

The only arenavirus that was relevant for this risk assessment was Junin virus, as the other viruses are present in countries that are not in the database [23] and/or no rodents were imported from these countries in 2013/2014 or, in case of Lymphocytic Choriomeningitis Virus (LCMV), the virus is already present in the Netherlands. Junin virus can be introduced in the Netherlands via import of infected rodents, that can shed the virus for a lifetime without developing disease themselves. Whether rodent species in the Netherlands are susceptible to Junin virus and can be part of a natural cycle in the Netherlands, is unknown. In 2013–2014, only nine rodents from countries where Junin virus is present were transited through the Netherlands and these were not the main reservoir species *Calomys musculinus*. However, Junin virus has been isolated from several rodent species and also hares, making the association of Junin virus with a single rodent species less definite than for other arenaviruses [40],[41]. Although the risk of exposure is considered very low, human impact of Junin virus infection can be considerable.

In the Netherlands, import control and registrations of exotic animals is done by the Netherlands Food and Consumer Product Safety Authority (NVWA). Import data are automatically transferred to TRACES (TRAde Control and Expert System), a web application of the European Commission connecting official veterinary services. This application makes it possible to trace back all imports of animals and enables quick response to any alert for better protection of animal and public health. However, the records of shipments of exotic animals in this database often only included a rough classification of the type of animals imported i.e. mammal, reptile, amphibian, bird or fish. More detailed information about numbers of animals and sometimes species had to be extracted from the attached (often handwritten) health certificates. For reptiles and amphibians, these health certificates are very basic trade documents concerning species, number and origin of animals without any obligatory testing of animals. Under the current legislation, it is not possible to perform routine microbiological testing of exotic animals upon arrival at Schiphol airport. However, this could be of great value for improvement of import control regulations and the risk assessment would be strengthened by the microbiological testing of exotic animals for zoonotic pathogens that are not yet covered by animal health legislation. The current risk analysis only assessed the public health risk of imports of live exotic animals into the Netherlands in the period of 2013 and 2014. Differences in trade flows and the epidemiological situation in the world can influence the public health risks. These differences were already observed between 2013 and 2014, although no trends could be extracted from this short period [23]. In the USA, the number of annual wildlife shipments has doubled over a period of thirteen years [2]. Whether this holds true for the Netherlands is unknown, but it shows the importance of updating this risk assessment regularly as trade patterns of exotic animals can change and also epidemiological situation and/or new information on the susceptibility of exotic animals to zoonotic pathogens can become available.

To get an indication of the illegal import of live exotic animal species and trade routes into the Netherlands via Schiphol airport, only e-mail correspondence between NVWA staff members and customs about seizures of illegally imported animals or animal products was available. An inventory was made of the numbers and species of exotic animals seized at Schiphol airport between January 2012 and December 2014. In 2015, an online registration system became available for recording seizures of illegally imported animals via Schiphol Airport. This electronic database for NVWA staff members, in which they can enter all necessary information concerning seizures of illegal animals and animal products, is the first step towards building a reliable database for further analysis.

Including additional data on illegal imports in this risk assessment could increase the risk for public health of the total exotic animal trade, as pathogens that were now excluded because no relevant animal species were imported, may be included when assessing the risk of illegal imports as well. The health status of illegally imported animals is furthermore unknown and may pose a much higher risk for public health than legally imported animals. The estimated illegal trade range of 1,700 to 245,000 illegally and transited animals per year shows the difficulty of estimating the numbers of illegally imported animals.

## Conclusion

So far, this is the first time the public health risk of legally imported live exotic animals into the Netherlands was assessed. This study showed that the overall public health risk of the introduction of exotic pathogens into the Netherlands via the legal import of exotic animals is considered low, which is supported by the lack of large scale outbreaks in the human population caused by exotic animals. However, due to changes in the epidemiological situation worldwide and changing trade patterns, this risk assessment needs regular updating. As this is very time-consuming, and not quantitative, an easily adaptable (quantitative) risk assessment tool should be developed, which can contribute to the early detection of high-risk trade flows of exotic animals in the future. The risk assessment tool can be strengthened by the incorporation of microbiological data. To start with, the large numbers of imported reptiles and amphibians should be risk based or at random sampled and tested for *Salmonella* spp. to enable better estimates of the risk of importing exotic animals infected with *Salmonella* spp. Illegally imported animals did not have a health check before exportation and are not accompanied by a health certificate; therefore, it is expected that the illegal imports pose a more substantial risk for public health than legally imported animals. Analysis of the seizure data of illegally imported animals via Schiphol Airport registered in the recently developed online registration system can contribute to risk-based checks of incoming flights and development of risk mitigation measures.

## Supporting information

S1 File Overview of references used within S1, S2 and S3 Tables. (DOCX)

S1 Table (DOCX)

S2 Table (DOCX)

S3 Table (DOCX)
