# Supplementary material for: Live exotic animals legally and illegally imported via the main Dutch airport and considerations for public health
Source: PLoS One. 2019 Jul 24;14(7):e0220122. doi: 10.1371/journal.pone.0220122 (PMC6655733; doi:10.1371/journal.pone.0220122)
Supplement: S1 File — (DOCX) [file pone.0220122.s001.docx]

**S4: References S1, S2, S3

General**

EmZOO, CDC, OIE – Wahid, Promed-mail
Pavlin, B.I., Schloegel, L.M., Daszak, P., 2009. Risk of importing zoonotic diseases through wildlife trade, United States. Emerging infectious diseases 15, 1721.
Travis, D. A., Watson, R. P., & Tauer, A., 2011. The spread of pathogens through trade in wildlife. Revue Scientifique et Technique-OIE 30, 219.

**Per pathogen**

Andes virus (ANDV)
- CDC (http://www.cdc.gov/hantavirus/index.html)

Argentine haemorrhagic fever/ Junin virus
- Public Health Agency of Canada (http://www.phac-aspc.gc.ca/lab-bio/res/psds-ftss/junin-eng.php)

Barmah Forest virus
- CDC factsheet (http://health.nt.gov.au/library/scripts/objectifyMedia.aspx?file=pdf/44/83.pdf&siteID=1&str_title=Barmah Forest virus.pdf)

Batai virus
- Reusken, C., De Vries, A., Ceelen, E., Beeuwkes, J., & Scholte, E. J. (2011). A study of the circulation of West Nile virus, Sindbis virus, Batai virus and Usutu virus in mosquitoes in a potential high-risk area for arbovirus circulation in the Netherlands,“De Oostvaardersplassen.”. Eur Mosq Bull, 29, 66-81.
- CDC (https://wwwn.cdc.gov/arbocat/VirusDetails.aspx?ID=52&SID=2)

Bayou virus (BAYV)
- CDC (http://wwwnc.cdc.gov/eid/article/4/1/pdfs/98-0115.pdf)

Bhanja virus
- CDC (https://wwwn.cdc.gov/arbocat/VirusDetails.aspx?ID=64&SID=1)

Black Creek Canal virus (BCCV)
- CDC (http://www.cdc.gov/hantavirus/index.html)

Bolivian haemorrhagic fever/ Machupo virus
- Public Health Agency of Canada (http://www.phac-aspc.gc.ca/lab-bio/res/psds-ftss/machupo-eng.php)

BSE prion
- CDC (http://www.cdc.gov/prions/bse/index.html)

Burkholdera mallei
- CFSPH (http://www.cfsph.iastate.edu/Factsheets/pdfs/glanders.pdf)

California encephalitis virus
- EmZoo (http://ezips.rivm.nl/pathogens/default.aspx?page=detail&patid=4067)

Cano Delgadito virus (CADV)
- Berger, S. A., Calisher, C. H., Keystone, J. S., & Leggat, P. A. (2004). Exotic Viral Diseases: a Global Guide. Journal of Travel Medicine, 11(3), 194-194.

Cercopithecine herpesvirus-1 (herpes B)
- CDC (http://www.cdc.gov/herpesbvirus/cause-incidence.html)

Choclo virus (CHOV)
- CDC (http://www.cdc.gov/hantavirus/index.html)

Cholera / Vibrio cholerae
- CDC (http://www.cdc.gov/cholera/index.html)

Colorado tick fever virus
- CDC (http://www.cdc.gov/coloradotickfever/index.html)

Crimean-Congo hemorrhagic fever virus
- CDC (http://www.cdc.gov/vhf/crimean-congo/resources/distribution-map.html)
- CDC (http://www.cdc.gov/vhf/crimean-congo/CCHF-FactSheet.pdf)
- Ergonul, O., 2012. Crimean–Congo hemorrhagic fever virus: new outbreaks, new discoveries. Current opinion in virology 2, 215-220.
- Široký, P., Bělohlávek, T., Papoušek, I., Jandzik, D., Mikulíček, P., Kubelová, M., & Zdražilová-Dubská, L. (2014). Hidden threat of tortoise ticks: high prevalence of Crimean-Congo haemorrhagic fever virus in ticks Hyalomma aegyptium in the Middle East. Parasit Vectors, 11, 101.

Dhori virus (Batken virus)
- CDC (https://wwwn.cdc.gov/arbocat/VirusDetails.aspx?ID=133&SID=1)

Dobrava-Belgrade virus (DOBV)
- Papa, A. (2012). Dobrava-Belgrade virus: Phylogeny, epidemiology, disease. Antiviral research, 95(2), 104-117.

Eastern equine encephalitis virus
- USGS National Wildlife Health Center (http://www.phac-aspc.gc.ca/lab-bio/res/psds-ftss/equine-eng.php)
- Public Health Agency of Canada (http://www.nwhc.usgs.gov/publications/field_manual/chapter_20.pdf)
- CDC (http://www.cdc.gov/EasternEquineEncephalitis/)
- OIE disease distribution map (http://www.oie.int/wahis_2/public/wahid.php/Diseaseinformation/Diseasedistributionmap)
- CFSPH (http://www.cfsph.iastate.edu/Factsheets/pdfs/easter_wester_venezuelan_equine_encephalomyelitis.pdf)

Echinococcus granulosus
- CDC (http://www.cdc.gov/parasites/echinococcosis/)
- Eckert, J., Thompson, R. C. A., Michael, S. A., Kumaratilake, L. M., & El-Sawah, H. M. (1989). Echinococcus granulosus of camel origin: development in dogs and parasite morphology. Parasitology Research, 75(7), 536-544.

Ehrlichia chaffeensis
- CDC (http://www.cdc.gov/ehrlichiosis/)
- EmZoo (http://ezips.rivm.nl/pathogens/default.aspx?page=detail&patid=4101)

Erve virus
- EmZoo (http://ezips.rivm.nl/pathogens/default.aspx?page=detail&patid=4070)

Francisella tularensis subspecies tularensis
- Petersen, J. M., Mead, P. S., & Schriefer, M. E. (2009). Francisella tularensis: an arthropod-borne pathogen. Veterinary research, 40(2), 1.

Hantaan virus (HTNV)
- CDC (http://www.cdc.gov/hantavirus/index.html)

Isla Vista virus (ISLAV)
- CDC (http://www.cdc.gov/hantavirus/index.html)

Japanese encephalitis virus
- CDC (http://www.cdc.gov/japaneseencephalitis/Maps/index.html)
- cfsph (http://www.cfsph.iastate.edu/Factsheets/pdfs/japanese_encephalitis.pdf)

Khabarovsk virus (KHAV)
- CDC (http://wwwnc.cdc.gov/eid/article/16/8/09-0470_article)

Laguna Negra virus (LANV)
- CDC (http://www.cdc.gov/hantavirus/index.html)

Lassa fever virus
- CDC (http://www.cdc.gov/vhf/lassa/)

Leprosy/Mycobacterium leprae
- CDC (http://www.cdc.gov/leprosy/)

Ljungan virus
- EmZoo (http://ezips.rivm.nl/pathogens/default.aspx?page=detail&patid=4070)

Louping ill virus
- CDC (http://www.cdc.gov/vhf/tbe/resources/TBE-FactSheet.pdf)
- CFSPH (http://www.cfsph.iastate.edu/Factsheets/pdfs/louping_ill.pdf)

Marburg virus
- CDC (http://www.cdc.gov/vhf/marburg/)

Menangle virus
- CFSPH (http://www.cfsph.iastate.edu/Factsheets/pdfs/menangle.pdf)

MERS coronavirus
- CDC (http://www.cdc.gov/coronavirus/mers/)

Monkey pox
- CDC (http://www.cdc.gov/poxvirus/monkeypox/)

Muleshoe virus (MULV)
- CDC (http://www.cfsph.iastate.edu/Factsheets/pdfs/hantavirus.pdf)

New York virus (NYV)
- CDC (http://www.cfsph.iastate.edu/Factsheets/pdfs/hantavirus.pdf)

Orienta tsutsugamushi
- CDC (http://wwwnc.cdc.gov/travel/yellowbook/2016/infectious-diseases-related-to-travel/rickettsial-spotted-typhus-fevers-related-infections-anaplasmosis-ehrlichiosis)

Prospect Hill virus (PHV)
- CDC (http://www.cfsph.iastate.edu/Factsheets/pdfs/hantavirus.pdf)

Rickettsia africae/ African tick bite fever
- Jensenius, M., Fournier, P. E., Kelly, P., Myrvang, B., & Raoult, D. (2003). African tick bite fever. The Lancet infectious diseases, 3(9), 557-564.
- CDC (http://wwwnc.cdc.gov/travel/diseases/african-tick-bite-fever)

Rickettsia conorii
- CDC (http://www.cdc.gov/otherspottedfever/)
- Levin, M. L., Killmaster, L. F., & Zemtsova, G. E. (2012). Domestic dogs (Canis familiaris) as reservoir hosts for Rickettsia conorii. Vector-Borne and Zoonotic Diseases, 12(1), 28-33.

Ricketssia prowazekii
- CFSPH (http://www.cfsph.iastate.edu/Factsheets/pdfs/typhus_fever.pdf)

Rickettsia typhii
- Eremeeva, M. E., Warashina, W. R., Sturgeon, M. M., Buchholz, A. E., Olmsted, G. K., Park, S. Y., ... & Karpathy, S. E. (2008). Rickettsia typhi and R. felis in rat fleas (Xenopsylla cheopis), Oahu, Hawaii. Emerging infectious diseases, 14(10), 1613.
- Zavala-Castro, J. E., Dzul-Rosado, K. R., Peniche-Lara, G., Tello-Martín, R., & Zavala-Velázquez, J. E. (2014). Isolation of Rickettsia typhi from Human, Mexico. Emerging infectious diseases, 20(8), 1411.
- Boostrom, A., Beier, M. S., Macaluso, J. A., Macaluso, K. R., Sprenger, D., Hayes, J., ... & Azad, A. F. (2002). Geographic association of Rickettsia felis-infected opossums with human murine typhus, Texas. Emerging infectious diseases, 8(6), 549-554.
- Maurin, M., & Raoult, D. (2002). Rickettsia typhi (murine typhus). Antimicrobial therapy and vaccine, 2nd edn. New York: Apple Trees Production, 200, 907-911.

Rickettsia rickettsia
- CDC (http://www.cdc.gov/rmsf/?rf=)

Rio Mamore virus (RIOMV)
- CDC (http://www.cfsph.iastate.edu/Factsheets/pdfs/hantavirus.pdf)

Rio Segundo virus (RIOSV)
- CDC (http://www.cfsph.iastate.edu/Factsheets/pdfs/hantavirus.pdf)

Rocio virus
- CDC (https://wwwn.cdc.gov/arbocat/VirusDetails.aspx?ID=401&SID=9)
- Henriques, D. F., Quaresma, J. A. S., Fuzii, H. T., Nunes, M. R. T., Silva, E. V. P. D., Carvalho, V. L., ... & Vasconcelos, P. F. D. C. (2012). Persistence of experimental Rocio virus infection in the golden hamster (Mesocricetus auratus). Memórias do Instituto Oswaldo Cruz, 107(5), 630-636.
- Medeiros, D. B., Nunes, M. R., Vasconcelos, P. F., Chang, G. J. J., & Kuno, G. (2007). Complete genome characterization of Rocio virus (Flavivirus: Flaviviridae), a Brazilian flavivirus isolated from a fatal case of encephalitis during an epidemic in Sao Paulo state. Journal of general virology, 88(8), 2237-2246.

Ross river virus
- CDC (https://wwwn.cdc.gov/arbocat/VirusDetails.aspx?ID=402&SID=1)
- Klapsing, P., MacLean, J. D., Glaze, S., McClean, K. L., Drebot, M. A., Lanciotti, R. S., & Campbell, G. L. (2005). Ross River virus disease reemergence, Fiji, 2003–2004. Emerging infectious diseases, 11(4), 613.
- Centre for disease control, Northern territory government (http://health.nt.gov.au/library/scripts/objectifyMedia.aspx?file=pdf/45/27.pdf&siteID=1&str_title=Ross%20River%20virus.pdf)

Saint Louis encephalitis virus
- CDC (http://www.cdc.gov/sle/)
- Public health agency Canada (http://www.phac-aspc.gc.ca/lab-bio/res/psds-ftss/st-louis-encephalit-eng.php#note17)

Salmonella spp.
- CDC (http://www.cdc.gov/salmonella/general/technical.html)
- Hoelzer, K., Switt, A.M., Wiedmann, M., 2011. Animal contact as a source of human non-typhoidal salmonellosis. Vet res 42, 34.
- Warwick, C., Lambiris, A., Westwood, D., Steedman, C., 2001. Reptile-related salmonellosis. Journal of the Royal Society of Medicine 94, 124.
- Whitten, T., Bender, J., Smith, K., Leano, F., Scheftel, J., 2015. Reptile‐Associated Salmonellosis in Minnesota, 1996–2011. Zoonoses and public health 62, 199-208.

SARS corona virus
- WHO (http://www.who.int/ith/diseases/sars/en/)

Simian foamy virus
- Calattini, S., Betsem, E. B. A., Froment, A., Mauclère, P., Tortevoye, P., Schmitt, C., ... & Gessain, A. (2007). Simian foamy virus transmission from apes to humans, rural Cameroon. Emerging infectious diseases, 13(9), 1314-20.

Sin Nombre virus (SNV)
- CDC (http://www.cdc.gov/hantavirus/)

Sindbis virus
- ECDC (http://ecdc.europa.eu/en/healthtopics/sindbis_fever/factsheet-health-professionals/Pages/factsheet-for-health-professionals.aspx)

South American hemorrhagic fever arenaviruses
- CDC (http://www.cdc.gov/vhf/virus-families/arenaviridae.html)
- Public health agency canada (http://www.phac-aspc.gc.ca/lab-bio/res/psds-ftss/junin-eng.php)

Taenia solium
- CDC (http://www.cdc.gov/parasites/taeniasis/)

Tahyna virus
- Lu, Z., Lu, X. J., Fu, S. H., Zhang, S., Li, Z. X., Yao, X. H., ... & Liang, G. D. (2009). Tahyna virus and human infection, China. Emerging infectious diseases, 15(2), 306.
- EmZoo (http://ezips.rivm.nl/pathogens/default.aspx?page=detail&patid=4074)

T-cell lymphotropic virus 1/HTLV-1
- Gonçalves, D. U., Proietti, F. A., Ribas, J. G. R., Araújo, M. G., Pinheiro, S. R., Guedes, A. C., & Carneiro-Proietti, A. B. F. (2010). Epidemiology, treatment, and prevention of human T-cell leukemia virus type 1-associated diseases. Clinical microbiology reviews, 23(3), 577-589.
- Centre for disease control, Northern territory government (http://health.nt.gov.au/library/scripts/objectifyMedia.aspx?file=pdf/45/19.pdf&siteID=1&str_title=HTLV-I%20.pdf)

Thailand virus (THAIV)
- Mackenzie, J. S. (Ed.). (1999). Hantaviruses: Emerging Viral Diseases. DIANE Publishing.

Thogotovirus (thogoto thogoto)
- CDC (https://wwwn.cdc.gov/Arbocat/VirusDetails.aspx?ID=477&SID=12)

Thottapalayam virus (TPMV)
- Song, J. W., Baek, L. J., Schmaljohn, C. S., & Yanagihara, R. (2007). Thottapalayam virus, a prototype shrewborne hantavirus. Emerging infectious diseases, 13(7), 980.

Tick-borne encephalitis virus
- CDC (http://www.cdc.gov/vhf/tbe/)

Topografov virus (TOPV)
- Clement, J., Heyman, P., McKenna, P., Colson, P., & Avsic-Zupanc, T. (1997). The hantaviruses of Europe: from the bedside to the bench. Emerging Infectious Diseases, 3(2), 205.

Toxoplasma gondii
- Sibley, L. D., Khan, A., Ajioka, J. W., & Rosenthal, B. M. (2009). Genetic diversity of Toxoplasma gondii in animals and humans. Philosophical Transactions of the Royal Society B: Biological Sciences, 364(1530), 2749-2761.

Tribec virus
- CDC (https://wwwn.cdc.gov/arbocat/VirusDetails.aspx?ID=489&SID=1)

Trichinella britovi
- CDC (http://www.cdc.gov/parasites/trichinellosis/)

Trichinella murrelli

- CDC (http://www.cdc.gov/parasites/trichinellosis/)
- Gottstein, B., Pozio, E., & Nöckler, K. (2009). Epidemiology, diagnosis, treatment, and control of trichinellosis. Clinical Microbiology Reviews, 22(1), 127-145.

Trichinella native
- CDC (http://www.cdc.gov/parasites/trichinellosis/)

Trichinella nelson
- CDC (http://www.cdc.gov/parasites/trichinellosis/)
- Gottstein, B., Pozio, E., & Nöckler, K. (2009). Epidemiology, diagnosis, treatment, and control of trichinellosis. Clinical Microbiology Reviews, 22(1), 127-145.

Trichinella papuae
- CDC (http://www.cdc.gov/parasites/trichinellosis/)

Trichinella pseudospiralis
- CDC (http://www.cdc.gov/parasites/trichinellosis/)
- Pozio, E. (2005). The broad spectrum of Trichinella hosts: from cold-to warm-blooded animals. Veterinary parasitology, 132(1), 3-11.

Trichinella zimbabwensis
- CDC (http://www.cdc.gov/parasites/trichinellosis/)

Venezuelan equine encephalitis virus
-CFSPH (http://www.cfsph.iastate.edu/FastFacts/pdfs/easter_wester_venezuelan_equine_encephalomyelitis_F.pdf)
- Public health agency of Canada (http://www.phac-aspc.gc.ca/lab-bio/res/psds-ftss/ven-encephalit-eng.php)
- Aguilar, P. V., Estrada-Franco, J. G., Navarro-Lopez, R., Ferro, C., Haddow, A. D., & Weaver, S. C. (2011). Endemic Venezuelan equine encephalitis in the Americas: hidden under the dengue umbrella. Future virology, 6(6), 721-740.

Wesselsbron virus
- CDC (https://wwwn.cdc.gov/Arbocat/VirusDetails.aspx?ID=518&SID=1)

West Nile virus
- OIE map 2014 jul-dec. and 2013 jul-dec
- CDC (http://www.cdc.gov/westnile/index.html)
- CDC (http://www.cdc.gov/westnile/resources/pdfs/birdspecies1999-2012.pdf)
- CFSPH (http://www.cfsph.iastate.edu/Factsheets/pdfs/west_nile_fever.pdf)
- WHO (http://www.who.int/mediacentre/factsheets/fs354/en/ )

Western equine encephalitis virus
- CFSPH (http://www.cfsph.iastate.edu/Factsheets/pdfs/easter_wester_venezuelan_equine_encephalomyelitis.pdf)
- Public health agency of canada (http://www.phac-aspc.gc.ca/lab-bio/res/psds-ftss/equine-eng.php)

Yersinia pestis
- CDC (http://www.cdc.gov/plague/transmission/index.html)

- Abbott, R.C., and Rocke, T.E., 2012, Plague: U.S. Geological Survey Circular 1372, 79 p.
- Hubálek, Z., & Rudolf, I. (2010). Microbial zoonoses and sapronoses. Springer Science & Business Media.
- Coutinho, E. M., de Almeida, A. M., & Almeida, C. R. D. (1982). Histopathology of Yersinia pestis infection in rodents from plague foci of Brazilian Northeast. Memórias do Instituto Oswaldo Cruz, 77(2), 139-151.
