# Supplementary material for: Live exotic animals legally and illegally imported via the main Dutch airport and considerations for public health
Source: PLoS One. 2019 Jul 24;14(7):e0220122. doi: 10.1371/journal.pone.0220122 (PMC6655733; doi:10.1371/journal.pone.0220122)
Supplement: S1 Table — (DOCX) [file pone.0220122.s002.docx]

**S1: Pathogen searches + first selection based on inclusion criteria**

**Table 1: EmZoo list 2010 (Havelaar et al., 2010)**

|  | **Zoonotic** | **Absent in NL** | **Absent on health certificate** | **Transmission live animals to humans (also via vector)** | **Vector is present** | **Conclusion** |
| --- | --- | --- | --- | --- | --- | --- |
| Influenza A virus (avian) H5N1 | yes | yes | no |  |  | Excluded |
| *Toxoplasma gondii* | yes | no* | yes | Yes | na | Included |
| Japanese encephalitis virus | yes | yes | yes | yes | yes | Included |
| *Campylobacter* spp. | yes | no |  |  |  | Excluded |
| *Mycobacterium bovis* | yes | yes | no |  |  | Excluded |
| BSE prion | yes | yes | yes | no |  | Excluded |
| *Coxiella burnetii* | yes | no |  |  |  | Excluded |
| *Anaplasma phagocytophila* | yes | no |  |  |  | Excluded |
| *Streptococcus suis* | yes | no |  |  |  | Excluded |
| *Leptospira interrogans* | yes | no |  |  |  | Excluded |
| West Nile virus | yes | yes | yes | yes | yes | Included |
| Crimean-Congo hemorrhagic fever virus | yes | yes | yes | yes | yes | Included |
| Dobrava-Belgrade virus | yes | yes | yes | yes | na | Included |
| Rabies virus (classic) | yes | yes | no |  |  | Excluded |
| *Yersinia pestis* | yes | yes | yes | yes | yes | Included |
| *Fasciola hepatica* | yes | no |  |  |  | Excluded |
| *Capnocytophaga canimorsus* | yes | no |  |  |  | Excluded |
| *Francisella tularensis* | yes | no* | yes | yes | yes | Included |
| Eastern equine encephalitis virus | yes | yes | yes | yes | yes | Included |
| *Chlamydophila psittaci* | yes | no |  |  |  | Excluded |
| Tick-borne encephalitis virus | yes | yes | yes | yes | yes | Included |
| *Staphylococcus aureus* (methicillin resistant) | yes | no |  |  |  | Excluded |
| Seoul virus | yes | no |  |  |  | Excluded |
| *Bartonella henselae* | yes | no |  |  |  | Excluded |
| European bat lyssa virus | yes | no |  |  |  | Excluded |
| *Brucella melitensis* | yes | yes | no |  |  | Excluded |
| *Mycobacterium avium* | yes | no |  |  |  | Excluded |
| Puumala virus | yes | no |  |  |  | Excluded |
| California encephalitis virus | yes | yes | yes | yes | yes | Included |
| *Brucella suis* | yes | yes | no |  |  | Excluded |
| Hepatitis E virus | yes | no |  |  |  | Excluded |
| Saint Louis encephalitis virus | yes | yes | yes | yes | yes | Included |
| *Salmonella* spp. | yes | no* | yes | yes | yes | Included |
| *Pasteurella multocida* | yes | no |  |  |  | Excluded |
| *Echinococcus multilocularis* | yes | no |  |  |  | Excluded |
| *Escheria coli* shiga toxin producing | yes | no |  |  |  | Excluded |
| *Chlamydophila abortus* | yes | no |  |  |  | Excluded |
| *Yersinia enterocolitica* | yes | no |  |  |  | Excluded |
| *Trichinella spp.* | yes | no* | yes | no |  | Excluded |
| *Clostridium botulinum* (toxins) | yes | no |  |  |  | Excluded |
| Monkeypox virus | yes | yes | yes**^**^** | yes | na | Included |
| *Rickettsia conorii* | yes | yes | yes | yes | yes | Included |
| Lymphocytic choriomeningitis virus | yes | no |  |  |  | Excluded |
| Western equine encephalitisvirus | yes | yes | yes | yes | yes | Included |
| *Rickettsia rickettsii* | yes | yes | yes | yes | yes | Included |
| Louping ill virus | yes | yes | yes | yes | yes | Included |
| *Cryptosporidium parvum* | yes | no |  |  |  | Excluded |
| Eyach virus | yes | no |  |  |  | Excluded |
| Cowpox virus | yes | no |  |  |  | Excluded |
| *Borrelia spp.* | yes | no |  |  |  | Excluded |
| *Cryptococus neoformans* var. *Gattii* | yes | no |  |  |  | Excluded |
| *Ascaris suum* | yes | no |  |  |  | Excluded |
| Rocio virus | yes | yes | yes | yes | yes | Included |
| *Leishmania spp.* | yes | yes | yes | Yes | no | Excluded |
| *Giardia lamblia* | yes | no |  |  |  | Excluded |
| *Toxocara canis/cati* | yes | no |  |  |  | Excluded |
| *Cryptococus neoformans* var. *Neoformans* | yes | no |  |  |  | Excluded |
| Ljungan virus | yes | yes | yes | yes | na | Included |
| *Babesia divergens/microti* | yes | no |  |  |  | Excluded |
| Tahyna virus | yes | yes | yes | yes | yes | Included |
| Tribec virus | yes | yes | yes | yes | yes | Included |
| *Taenia solium* | yes | yes | yes | yes | na | Included |
| *Baylisascaris procyonis* | yes | no |  |  |  | Excluded |
| *Rickettsia helvetica* | yes | no |  |  |  | Excluded |
| *Rickettsia spp.* | yes | yes | yes | yes | yes | Included |
| *Clostridium difficile* | yes | no |  |  |  | Excluded |
| Venezuelan equine encephalitis virus | yes | yes | yes | yes | yes | Included |
| Colorado tick fever virus | yes | yes | yes | yes | yes | Included |
| *Echinococcus granulosus* | yes | yes | yes | yes | yes | Included |
| *Anisakis simplex* | yes | no |  |  |  | Excluded |
| Orf virus | yes | no |  |  |  | Excluded |
| Erve virus | yes | yes | yes | yes | unknown | Included |
| *Taenia saginata* | yes | no |  |  |  | Excluded |
| *Erysipelothrix rhusiopathiae* | yes | no |  |  |  | Excluded |
| Batai virus | yes | yes | yes | yes | yes | Included |
| *Ehrlichia chaffeensis* | yes | yes | yes | yes | yes | Included |
| *Fasciola hepatica* | yes | no |  |  |  | Excluded |
| Bhanja virus | yes | yes | yes | yes | yes | Included |
| Sindbis virus | yes | yes | yes | yes | yes | Included |
| *Burkholderia mallei* | yes | yes | yes | yes | na | Included |
| *Dirofilaria immitis/repens* | yes | no |  |  |  | Excluded |
| Thogotovirus | yes | yes | yes | yes | yes | Included |
| Barmah Forest virus | yes | yes | yes | yes | yes | Included |
| Wesselsbron virus | yes | yes | yes | yes | yes | Included |
| Ross river virus | yes | yes | yes | yes | yes | Included |
| Dhori virus (Batken virus) | yes | yes | yes | yes | yes | Included |

Empty cells: As soon as a pathogen does not meet an inclusion criterium (starting with the zoonotic potential) it is excluded and not checked for the remaining inclusion criteria.

* These pathogens are present in the Netherlands, but more virulent strains can be introduced by imported animals from e.g. South America.
^**^ Does not cover all animal species

Na: not relevant

**Table 2: ProMed list + WAHID database***

|  | **Zoonotic** | **Absent in NL** | **Absent on health certificate** | **Transmission live animals to humans (also via vector)** | **Vector is present** | **Conclusion** |
| --- | --- | --- | --- | --- | --- | --- |
| **USA** |  |  |  |  |  |  |
| Listeriosis = *Listeria monocytogenes* | yes | no |  |  |  | Excluded |
| Murine Typhus = *rickettsia typhi* | yes | yes | yes | yes | yes | Included |
| Tickborne relapsing fever = *Borrelia spp.* | yes | yes | yes | yes | no | Excluded |
| Rat bite fever = *Streptobacillus moniliformis* and *Spirillum minor* | yes | no |  |  |  | Excluded |
| Tularemia = *Francisella tularensis* | yes | no |  |  |  | Excluded |
| **Indonesia** |  |  |  |  |  |  |
| Ebolavirus | yes | yes | no |  |  | Excluded |
| Anthrax = *Bacillus anthracis* | yes | yes | no |  |  | Excluded |
| Dengue virus | yes | yes | yes | yes | no | Excluded |
| **Vietnam** |  |  |  |  |  |  |
| **Tanzania** |  |  |  |  |  |  |
| **El Salvador** |  |  |  |  |  |  |
| *Trypanosoma cruzi* | yes | yes | yes | yes | no | Excluded |
| **Togo** |  |  |  |  |  |  |
| **Suriname** |  |  |  |  |  |  |
| **Egypt** |  |  |  |  |  |  |
| MERS coronavirus | yes | yes | yes | yes | na | Included |
| **Guyana** |  |  |  |  |  |  |
| **Canada** |  |  |  |  |  |  |
| **Uganda** |  |  |  |  |  |  |

** The WAHID database did not provide us with any additional pathogens that were not considered yet*Empty cells: As soon as a pathogen does not meet an inclusion criterium (starting with the zoonotic potential) it is excluded and not checked for the remaining inclusion criteria.

Na: not relevant

**Table 3: Literature and expert opinion**

|  | **Zoonotic** | **Absent in NL** | **Absent on health certificate** | **Transmission live animals to humans (also via vector)** | **Vector is present** | **Conclusion** |
| --- | --- | --- | --- | --- | --- | --- |
| **Pavlin, 2009 [19]** |  |  |  |  |  |  |
| Cercopithecine herpesvirus-1 (herpes B) | yes | yes | yes | yes | na | Included |
| Lassa fever virus | yes | yes | yes | yes | na | Excluded* |
| Marburg virus | yes | yes | yes | yes | na | Included |
| South American hemorrhagic fever/arenaviruses | yes | yes | yes | yes | na | Included |
| Hantaviruses associated with HCPS | yes | yes | yes | yes | na | Included |
| SARS corona virus | yes | yes | yes | yes | na | Included |
| Yellow fever virus | yes | yes | yes | yes | no | Excluded |
| **Travis, 2011** |  |  |  |  |  |  |
| *Rickettsia africae*/ African tick bite fever | yes | yes | yes | yes | yes | Included |
| Argentine haemorrhagic fever/ Junin virus | yes | yes | yes | yes |  | Excluded* |
| Bolivian haemorrhagic fever/ Machupo virus | yes | yes | yes | yes |  | Excluded* |
| Cholera / *Vibrio cholerae* | yes | yes | yes | no |  | Excluded |
| *Ehrlichia canis* / ehrlichiosis | yes | no |  |  |  | Excluded |
| Leprosy/*Mycobacterium leprae* | yes | yes | yes | yes | na | Included |
| Menangle virus | yes | yes | yes | yes | na | Included |
| *Neospora caninum* | yes | no |  |  |  | Excluded |
| Phocine distemper virus | yes | no |  |  |  | Excluded |
| Simian foamy virus | yes | yes | yes | yes | na | Included |
| T-cell lymphotropic virus 1/HTLV-1 | yes | yes | yes | yes | na | Included |
| **Expert opinion** |  |  |  |  |  |  |
| Andes virus (ANDV) | yes | yes | yes | yes | na | Included |
| Bayou virus (BAYV) | yes | yes | yes | yes | na | Included |
| Black Creek Canal virus (BCCV) | yes | yes | yes | yes | na | Included |
| Cano Delgadito virus (CADV) | yes | yes | yes | yes | na | Included |
| Choclo virus (CHOV) | yes | yes | yes | yes | na | Included |
| Hantaan virus (HTNV) | yes | yes | yes | yes | na | Included |
| Isla Vista virus (ISLAV) | yes | yes | yes | yes | na | Included |
| Khabarovsk virus (KHAV) | yes | yes | yes | yes | na | Included |
| Laguna Negra virus (LANV) | yes | yes | yes | yes | na | Included |
| Muleshoe virus (MULV) | yes | yes | yes | yes | na | Included |
| New York virus (NYV) | yes | yes | yes | yes | na | Included |
| Prospect Hill virus (PHV) | yes | yes | yes | yes | na | Included |
| Puumala virus (PUUV) | yes | no |  |  |  | Excluded |
| Rio Mamore virus (RIOMV) | yes | yes | yes | yes | na | Included |
| Rio Segundo virus (RIOSV) | yes | yes | yes | yes | na | Included |
| Seoul virus (SEOV) | yes | no |  |  |  | Excluded |
| Sin Nombre virus (SNV) | yes | yes | yes | yes | na | Included |
| Thailand virus (THAIV) | yes | yes | yes | yes | na | Included |
| Thottapalayam virus (TPMV) | yes | yes | yes | yes | na | Included |
| Topografov virus (TOPV) | yes | yes | yes | yes | na | Included |
| Tula virus (TULV) | yes | no |  |  |  | Excluded |
| *Trichinella spiralis* | yes | no |  |  |  | Excluded |
| *Trichinella pseudospiralis* | yes | no |  |  |  | Excluded |
| *Trichinella nativa* | yes | yes | yes | no** |  | Excluded |
| *Trichinella nelson* | yes | yes | yes | no** |  | Excluded |
| *Trichinella britovi* | yes | no |  |  |  | Excluded |
| *Trichinella murrelli* | yes | yes | yes | no** |  | Excluded |
| *Trichinella papuae* | yes | yes | yes | no |  | Excluded |
| *Trichinella zimbabwensis* | yes | yes | yes | no |  | Excluded |

Empty cells: As soon as a pathogen does not meet an inclusion criterium (starting with the zoonotic potential) it is excluded and not checked for the remaining inclusion criteria.
* Included as: South American hemorrhagic fever arenaviruses
** *Trichinella spp*. can be present in muscles of live imported exotic animals, but since meat inspection is mandatory of all susceptible animals intended for human consumption in the EU (EU Reg 1375/2015), the Trichinella transmission to humans is considered absent.

Na: not relevant
