# Supplementary material for: Live exotic animals legally and illegally imported via the main Dutch airport and considerations for public health
Source: PLoS One. 2019 Jul 24;14(7):e0220122. doi: 10.1371/journal.pone.0220122 (PMC6655733; doi:10.1371/journal.pone.0220122)
Supplement: S2 Table — (DOCX) [file pone.0220122.s003.docx]

**S2: Selection based on geographical spread and animal species that can carry the pathogen.**

**Table 1: Selection based on geographical spread and animal species that can carry the pathogen.**

| **Zoonotic pathogen** | **Geographical spread of pathogen** | **Were animals imported from these countries?** | **Animals that can carry the pathogen** | **Were these animals imported?** | **Conclusion** |
| --- | --- | --- | --- | --- | --- |
| Andes virus (ANDV) | South America | yes (Argentina, Chile, Uruguay) | Long-tailed pygmy rice rat (*Oligoryzomys longicaudatus*), shrews (family: Soricidae) , moles | Oligoryzomys longicaudatus from the : Muridae family were imported, but these were from Egypt. Only Dasyproctidae and Caviidae are imported from Argentina, which not known for carrying ANDV. | Excluded |
| Barmah Forest virus | Australia | no |  |  | Excluded |
| Batai virus | Europe, Malaysia; India; Thailand; Czechoslovakia; Soviet Ukraine, USSR; Cambodia. | yes, Russia | Birds | No birds were imported from Russia | Excluded |
| Bayou virus (BAYV) | United states and Canada | yes | Rice rat (*Oryzomys palustris*) | no | Excluded |
| Bhanja virus | India, Italy, Nigeria, Cameroon, Senegal, Yugoslavia, Central African Republic, USSR, Bulgaria, Somalia | yes, Russia | Sheep, goat, cattle, dogs | no | Excluded |
| Black Creek Canal virus (BCCV) | USA: Florida | yes | Cotton rat (*Sigmodon hispidus*) | no | Excluded |
| *Burkholdera mallei* | Africa, Asia, Middle East, Central America, South America |  | Horses, mules, donkeys, | no | Excluded |
| California encephalitis virus | USA: California | yes | Rodents, rabbits | No, only rodents from Argentina and Egypt were imported | Excluded |
| Cano Delgadito virus (CADV) | Venezuela | no |  |  | Excluded |
| Cercopithecine herpesvirus-1 (herpes B) | Worldwide (zoos) | yes | Macaques (rhesus (*Macaca mulatta*) and cynomolgus (*Macaca fascicularis*) | no | Excluded |
| Choclo virus (CHOV) | North and South America | yes | Rodents (pygmy rice rat (*Oligoryzomys fulvescens*)) | Only Dasyproctidae and Caviidae were imported from Argentina, not known for carrying Choclo virus | Excluded |
| Colorado tick fever virus | Western United States or western Canada. CTF was specifically reportable in six states: Arizona, Colorado, Montana, Oregon, Utah, and Wyoming. | yes | Small rodents such as squirrels, chipmunks, and mice | No, only rodents from Argentina and Egypt were imported | Excluded |
| Crimean-Congo hemorrhagic fever virus | China (xinjiang), Egypt, Kenya, Russia, Saudi Arabia, South Africa, Tanzania, Uganda, UAE | yes | Mechanical vector: birds as they carry the ticks. Amplifying hosts: Livestock (cattle, goats, sheep), hares. Other possible hosts: Tortoises (Testudines), rodents | yes | Included |
| Dhori virus (Batken virus) | India, Egypt, USSR, Portugal | yes (Egypt, Russia) | Camels, horses, goats, birds | Yes, these species were imported, but not from Egypt and Russia | Excluded |
| Dobrava-Belgrade virus (DOBV) | Europe (Balkan) | no |  |  | Excluded |
| Eastern equine encephalitis virus | USA, Canada, Colombia, Venezuela, Brazil, Ecuador, Costa Rica, Mexico, Georgia, Turkmenistan, Moldova | Yes USA, Canada, Colombia, Brazil, Ecuador, Mexico | Horses (no risk, dead end host), (wild) birds, Amphibians and reptiles are a possible reservoir for the virus to overwinter. | Yes, birds, amphibians and reptiles were imported from the USA and Canada. Amphibians were imported from Colombia. | Included |
| *Echinococcus granulosus* | Africa, Europe, Asia, the Middle East, Central and South America, and in rare cases, North America. | yes | Canines, cattle, sheep, camels, pigs | Yes, Lama and Vicunga were imported from Chile | Included |
| *Ehrlichia chaffeensis* | USA | yes | White-tailed deer, dogs, cats, coyotes, goats, deer, rodents | No, felidae and rodents were imported but not from the USA | Excluded |
| Erve virus | France | no |  |  | Excluded |
| *Francisella tularensis* subspecies tularensis | Noord Amerika | yes | Lagomorphs (rabbits and hares), | no | Excluded |
| Hantaan virus (HTNV) | Asia (Korea) | yes | *Apodemus agrarius* (the striped field mouse) | No rodents were imported from Asia | Excluded |
| Hantaviruses associated with HPCS | --> Excluded because species of interest of the genus Hantavirus are included. | | | | Excluded |
| Isla Vista virus (ISLAV) | North America | yes | *Microtus californicus* (Californian meadow vole) | No rodents were imported from North America | Excluded |
| Japanese encephalitis virus | Asia and western pacific | yes: China, Indonesia, Japan, Philippines, Russia, Singapore, Vietnam | The Japanese encephalitis virus causes disease in horses, donkeys, pigs and humans. This virus can also infect other domesticated animals including cattle, sheep, goats, dogs and cats, as well as wild mammals, reptiles, amphibians and birds; these infections are typically asymptomatic. Birds including herons and egrets, and swine are the most important maintenance hosts | Birds were imported from Singapore and the Philippines. Amphibians were imported from China, Indonesia and Vietnam. Reptiles from China, Indonesia, Philippines and Vietnam. Mammals were imported from Indonesia, Japan, Russia, Singapore. | Included |
| Khabarovsk virus (KHAV) | Russia | yes | *Microtus maximowiczii* voles | No rodents were imported from Russia | Excluded |
| Laguna Negra virus (LANV) | Paraguay, Argentina | yes | Vesper mouse, *Calomys laucha*, large vesper mouse *Calomys callosus* | 6 Dasyprocta and 3 Hydrochoerus were imported from Argentina | Excluded |
| Leprosy/*Mycobacterium leprae* | Angola, Brazil, Central African Republic, Democratic republic of congo, federated states of micronesia, India, Kiribati, Madagascar, Mozambique, Nepal. Republic of Marshall Islands, United republic of Tanzania. | Yes Brazil, Tanzania | Armadillo | no | Excluded |
| Ljungan virus | Sweden, Denmark, USA, UK | Yes USA | Several species of voles and lemmings | No only rodents were imported from Argentina and Egypt | Excluded |
| Louping ill virus | UK | no |  |  | Excluded |
| Marburg virus | Africa: Uganda, Zimbabwe, the Democratic Republic of the Congo, Kenya, Angola, and South Africa | Yes Kenya, South Africa | African fruit bat, *Rousettus aegyptiacus* | No | Excluded |
| Menangle virus | Australia | no |  |  | Excluded |
| MERS coronavirus | Saudi Arabia, United Arab Emirates, Qatar, Oman, Jordan, Kuwait, Yemen, Lebanon, Iran | Yes United Arab emirates, | Camels | Only reptiles and birds were imported from the United Arab emirates | Excluded |
| Monkey pox | subsaharan Africa | yes | African rodents, mangabeys | Mangabeys were imported from Ghana but they are tested for Monkey pox. | Excluded |
| Muleshoe virus (MULV) | North America |  | *Sigmodon hispidus* (cotton rat) | No rodents were imported from North America | Excluded |
| New York virus (NYV) | the northeastern US | Yes USA | *Peromyscus maniculatus* (deer mouse); *P. leucopus* (white-footed mouse) | No rodents were imported from the USA | Excluded |
| Prospect Hill virus (PHV) | USA | yes | Meadow vole | No rodents were imported from the USA | Excluded |
| *Rickettsia africae*/ African tick bite fever | South Africa, Botswana, Zimbabwe, Tanzania, Kenya, Burundi, Sudan, Ethiopia, Central African Republic, Gabon, Mali, Niger, Côte d'Ivoire, and Gambia, and on Guadeloupe in the French West Indies. | Yes South Africa and Tanzania | Cattle, goats, wild ungulates | Yes, Artiodactyla were imported from South Africa | Included |
| *Rickettsia conorii* | Mostly in mediterranean countries (Spain, Italy, Israel), but also (Sub Saharan) Africa, India, Southwest Asia | Yes Egypt, United Arab emirates, Ghana, Togo, Kenya, Tanzania, South Africa | *R sanguineus* (the brown dog tick) is the most common vector for *R. conorii*. Although these ticks can feed on a variety of mammalian and even avian animals, it is most closely associated with canines and primarily with the domestic dog, *Canis familiaris*. Not known for sure what the best reservoir host is. | No | Excluded |
| *Ricketssia spp.* | --> Not all subspecies of Rickettsia are included as there are too many, only subspecies listed in the EmZoo list or found in literature or Promed/Wahid were included. | | | | Excluded |
| *Rickettsia typhii* | Tropical and subtropical areas worldwide (Australia, china, Greece, USA, Mexico, Israel, Kuwait, Thailand, Hawaii) | yes | Commensal rodents (mainly *Rattus norvegicus*, and *Rattus rattus*) are considered the main reservoir of bacteria, but other vertebrate hosts may serve as reservoir including house mice, shrews, opossums, skunks, and cats. | Rodents were imported from Argentina and Egypt. Felidae from Egypt, South Africa, Tanzania, Indonesia, Singapore, Cuba, Mexico | Included |
| *Rickettsia rickettsii* | North and South America | Yes USA, Argentina etc. | Rodents and lagomorphs | Rodents (6 Dasyprocta and 3 Hydrochoerus) were imported from Argentina | Included |
| Rio Mamore virus (RIOMV) | Bolivia, Peru, Brazil | Yes Peru and Brazil | *Oligoryzomys microtis* | No only rodents were imported from Argentina and Egypt | Excluded |
| Rio Segundo virus (RIOSV) | Costa Rica | no | *R. mexicanus* |  | Excluded |
| Rocio virus | Brazil | yes | Wild birds | Birds (Psittacidae) were imported from Brazil | Included |
| Ross river virus | RRV is found throughout Australia, Papua New Guinea, parts of Indonesia and the western Pacific Islands. | Yes Indonesia | Macropods (kangaroos and wallabies) are thought to act as reservoirs but other species, including possums, horses, dogs, cats and bats can also be infected and may potentially act as amplifying hosts | Panthera and Petaurus (sugar glider) were imported from Indonesia. | Included |
| Saint Louis encephalitis virus | USA | yes | Bats, wild birds, domesticated fowl, killer whale, rodents, and possibly other mammals. Primary reservoirs are wild birds, domestic fowl and bats. | yes | Included |
| *Salmonella* spp. | Worldwide | yes | Salmonella live in the intestinal tracts of humans and other animals, including poultry and other birds, amphibians, and reptiles. Reptiles, such as turtles, lizards, and snakes, are particularly likely to harbor Salmonella | yes | Included |
| SARS corona virus | China, Other countries/areas in which chains of human-to-human transmission occurred after early importation of cases were Toronto in Canada, Hong Kong Special Administrative Region of China, Chinese Taipei, Singapore, and Hanoi in Viet Nam. | Yes, China, Canada, Singapore, Vietnam | Civet in China, uncertain, probably bats | no | Excluded |
| Simian foamy virus | Central Africa (democratic republic of the Congo, Cameroon, Gabon), Asia (Indonesia) | Yes Indonesia | Primates | no | Excluded |
| Sin Nombre virus (SNV) | North America |  | Deer mouse | No rodents were imported from North America | Excluded |
| Sindbis virus | SINV is found in Eurasia, Africa, and Australia but clinical SINV infections occur mostly in Northern Europe | Yes, Kenya, Uganda, Tanzania, South Africa, Egypt, Philippines | Birds, sporadically rodents, bats, amphibians have been implicated | Amphibians and birds were imported from Tanzania, birds were imported from South Africa and the Philippines, rodents were imported from Egypt | Included |
| South American hemorrhagic fever arenaviruses | Machupo in Bolivia, Junin in Argentina, Guanarito in Venezuela, Lassa in west Africa, Lujo in Zambia, Chapare in Bolivia, Sabia in Brazil | Yes, Argentina, West Africa (Togo and Ghana), Brazil | Rodents | yes | Included |
| *Taenia solium* | Higher rates of illness have been seen in people in Latin America, Eastern Europe, sub-Saharan Africa, India, and Asia. | yes | Pigs | no | Excluded |
| Tahyna virus | Europe, with exception of Benelux and Denmark. Asia (China) | China | Pigs, bats, rodents | Yes, but from China only amphibia and reptiles were imported | Excluded |
| T-cell lymphotropic virus 1/HTLV-1 | Japan, Africa, the Caribbean islands, and Central and South America emerging as the areas of highest prevalence in the world | yes | Primates (red colobus, sooty mangabeys etc.) | Yes, primate were imported from Peru, Ghana, Tanzania | Included |
| Thailand virus (THAIV) | Thailand | no | Murinae rodents |  | Excluded |
| Thogotovirus (thogoto thogoto) | Egypt, Kenya, Nigeria, Central African Republic , Sicily, Iran, Ethiopia, Cameroon, Portugal, Uganda | Yes, Egypt, Kenya, Uganda | Cattle, camels | no | Included |
| Thottapalayam virus (TPMV) | Asia (India, China) | Yes, China | shrews | Only reptiles and amphibia were imported from China | Excluded |
| Tick-borne encephalitis virus | parts of Europe, the Former Soviet Union, and Asia (China, Japan, Kazakhstan, Kyrgyzstan, Mongolia, and South Korea.) | Yes Russia, China, Japan | Rodents | No rodents were imported from Russia, China, Japan | Excluded |
| Topografov virus (TOPV) | Siberia | no | Lemmings | No | Excluded |
| *Toxoplasma gondii* | worldwide | yes | Felidae and lama | No felidae were imported from South America in the database. Imported lamas were not considered a risk because the chance that lama meat will be eaten by cats in the Netherlands was considered negligible. | Excluded |
| Tribec virus | Czechoslovakia, Rumania, Italy, USSR | Yes Russia | small rodents | No small rodents were imported from Russia | Excluded |
| Venezuelan equine encephalitis virus | South and Central America (Colombia, Costa Rica, Venezuela, Ecuador, Peru, French Guiana), Mexico, and the USA | Yes, Colombia, Ecuador, Peru, Mexico, USA | Equids (Horses, donkey, zebra), sylvatic rodents and wild birds | Birds from the USA and Peru | Included |
| Wesselsbron virus | South Africa, Zimbabwe, Senegal, Nigeria, Kenya, Cameroon, Central African Republic, Thailand. | Yes, South Africa, Kenya | Cattle, sheep, rodent | no | Excluded |
| West Nile virus | Africa, Europe, the Middle East, west and central Asia, and North America, Argentina | Yes, Africa (South Africa), middle East (Egypt, Saudi Arabia, United Arab Emirates), North America (USA), Argentina | Birds | Yes birds were imported from Tanzania, USA, United Arab Emirates, South Africa, Argentina | Included |
| Western equine encephalitis virus | North and South America | yes | Horses (no risk, dead end host), (wild) birds, Amphibians and reptiles are a possible reservoir for the virus to overwinter. | Yes, birds, amphibians and reptiles were imported from the USA, Suriname and Peru. Birds and reptiles were imported from Argentina. Birds were imported from Brazil. Amphibians were imported from Colombia, Ecuador. Reptiles and amphibians were imported from Guyana. | Included |
| *Yersinia pestis* | Africa, Asia, South America | yes | Rodents (domestic rats, squirrels), cats, | Rodents were imported from Argentina and Egypt. Felidae were imported from Egypt, South Africa, Tanzania, Indonesia, Singapore | Included |
