# Supplementary material for: Live exotic animals legally and illegally imported via the main Dutch airport and considerations for public health
Source: PLoS One. 2019 Jul 24;14(7):e0220122. doi: 10.1371/journal.pone.0220122 (PMC6655733; doi:10.1371/journal.pone.0220122)
Supplement: S3 Table — (DOCX) [file pone.0220122.s004.docx]

**S3: Risk assessment

Table 1: Risk assessment**

| **Zoonotic disease** | **Infection facts** | **Countries where pathogen is present and animals were imported/transited from** | **Specific animal species** | **Number and order/family of susceptible animals in database** | **Import or transit?** | **Impact** | **Summary** | **Expert 1** | **Expert 2** | **Expert 3** | **Expert 4*** |
| --- | --- | --- | --- | --- | --- | --- | --- | --- | --- | --- | --- |
| Arenaviruses | Family of viruses that are generally associated with rodent-transmitted diseases in humans. Arenaviruses that cause mild or severe illness in humans are LCMV, Junin, machupo, chapare, Guanarito, Lassa, Lujo and Sabia. | Argentina | Rodents | Argentina: Rodents Dasyprocta (6) and Hydrochoerus (3) | Transit 3rd countries | Clinical disease in 80% of the human infections. Three phases of disease: prodromal, neurological-hemorrhagic, and convalescence. Prodromal phase: flu-like symptoms, muscle pain, nausea and dizziness. Twenty to 30% of the patients develop neurologic and/or hemorrhagic symptoms: vomiting blood, nose bleeds, mental confusion, convulsions etc. The third phase (prolonged convalescence of one to three months): weakness, memory loss and irritability. Case fatality rate of viral haemorrhagic fever is 10-30%, which is lowered to 1% when treated with convalescent serum. However, 10% of the patients that receive this treatment, develop neurological problems | Of all arenaviruses that can cause disease in humans, only Junin virus could have been introduced. The other viruses are from countries that are not in the database or no rodents are imported from these countries. Risk from 9 rodents that were all in transit. Not known if these rodent species are also hosts for Junin virus. Disease can be lethal. | 1 | 2 or 3 | 2 | - |
| Crimean-Congo hemorrhagic fever virus | Hyalomma ticks are the principal source of human infection. Humans become infected through the bites of ticks, by contact with a patient with CCHF during the acute phase of infection, or by contact with blood or tissues from viraemic livestock. | China (Xinjiang), Egypt, Kenya, Saudi Arabia, South Africa, Tanzania, UAE | Mechanical vector: birds as they carry the ticks. Other possible hosts: Tortoises (Testudines) and rodents. A high prevalence (30.2%) of CCHFV was found in *Hyalomma aegyptium* ticks, removed from wild spur-thighed tortoises (Testudo graeca) in Turkey and Syria (Široký et al., 2014). The role of these tortoises in the transmission of CCHFV is unknown. It is also unknown whether testudines can serve as a reservoir of CCHFV in natural circulation or can only carry infected ticks. | China (only Hongkong in database, different region than Xinjiang): Testudines (204). Egypt: Rodentia (217). Kenya: Testudines (878).Russia: / Saudi Arabia: Testudines (4). South Africa: birds Ramphastidae (6). Tanzania: Many different bird species, 20 different families (391), Testudines (6). UAE: birds Charadriiformes (6) | China: 157 testudines import/47 testudines transit. Egypt: all rodents import. Kenya: all testudines in transit to Germany. Saudi Arabia: all testudines in transit to the UK. South Africa: all Ramphastidae import. Tanzania: 105 birds import/286 birds transit third countries, 6 testudines transit Czech republic. UAE: all birds in transit to Germany | CCHF has a sudden onset with symptoms including headache, high fever, back pain, joint pain, stomach pain, and vomiting. Red eyes, a flushed face, a red throat, and petechiae (red spots) on the palate are common. Symptoms may also include jaundice, and in severe cases, changes in mood and sensory perception. After four days of illness, other symptoms as large areas of severe bruising, severe nosebleeds, and uncontrolled bleeding at injection sites can be seen. CCHF is a severe disease with a high case fatality rate ranging from 9 to 50% | Risk from 403 birds (111 import/292 transit) and maybe 1092 testudines (170 import/922 transit) and 217 rodents (217 import). Birds can be a direct risk for humans. Should keep an eye on research into the role of testudines in CCHFV. Not much reported about rodents and CCHFV. Most known human cases are because of livestock. Disease can be severe, high fatality rates possible. | 3 | 2/3 | 2 | 2 |
| Eastern equine encephalitis virus | Eastern equine encephalitis virus (EEEV) is maintained in a cycle between *Culiseta melanura* mosquitoes and avian hosts in freshwater hardwood swamps. *Cs. melanura* is not considered to be an important vector of EEEV to humans because it feeds almost exclusively on birds. Transmission to humans requires mosquito species capable of creating a "bridge" between infected birds and uninfected mammals such as some *Aedes*, *Coquillettidia*, and *Culex* species. | USA, Canada, Colombia, Brazil, Ecuador, Mexico | Passerine birds are thought to be the principal reservoir hosts for North American EEEV in natural cycles, but small mammals (e.g., rodents) might also amplify the virus. The primary reservoir hosts for Madariaga virus (South American EEEV) are still uncertain, but small mammals might play a more prominent role. Some experiments have suggested that reptiles (especially snakes) might help maintain EEEV over the winter. Domesticated mammals including equids are not important in virus amplification. | USA: birds Ramphastidae (9) Phasianidae (7) Apterygidae (1), amphibians Anura (2322) Urodela (307), reptiles Squamata (6375) Testudines (167885). Canada: birds Phoenicopteridae (7) Falconidae (3), amphibians Anura (1036), reptiles Squamata (457). Colombia: amphibians Anura (88). Brazil: birds psittacidae (8). Ecuador: Amphibians Anura (92). Mexico: /. | USA: birds 1 apterygidae import/9 ramphastidae import/7 phasianidae transit; amphibians 2302 Anura import/20 Anura transit and 307 Urodela import; Reptiles 6367 squamata import/8 squamata transit and 158885 testudines import/9000 transit . Canada: birds 7 phoenicopteridae transit /3 falconidae transit; amphibians 1036 Anura import; Reptiles 390 squamata import/67 squamata transit. Colombia: amphibians 88 Anura import. Brazil: birds 8 psittacidae transit. Ecuador 60 Anura import/32 Anura transit. | EEEV infection can result in one of two types of illness, systemic or encephalitic Systemic infection has an abrupt onset and is characterized by chills, fever, malaise, arthralgia, and myalgia. The illness lasts 1 to 2 weeks, and recovery is complete when there is no central nervous system involvement. Encephalitic: Approximately a third of all people with EEE die from the disease. Death usually occurs 2 to 10 days after onset of symptoms but can occur much later. Of those who recover, many are left with disabling and progressive mental and physical sequelae, which include can range from minimal brain dysfunction to severe intellectual impairment, personality disorders, seizures, paralysis, and cranial nerve dysfunction. Many patients with severe sequelae die within a few years. | Risk from 36 birds (10 import), birds are the main reservoir host and mainly passerine birds: none of the 36 birds are passerine birds. No direct transmission between birds and humans. Many reptiles (6832 squamata and 167885 testudines) are imported and no rodents. Unknown what the risk is of reptiles. And amphibians also unknown (3538 Anura and 307 Urodela). For transmission to humans are mosquitoes needed. In case of encephalitic type of illness: high fatality rate. | 2 | Unknown** | 2 | 2 |
| *Echinococcus granulosus* | The parasite is transmitted to dogs when they ingest the organs of other animals that contain hydatid cysts. The cysts develop into adult tapeworms in the dog. Infected dogs shed tapeworm eggs in their feces which contaminate the ground. Sheep, cattle, goats, and pigs ingest tapeworm eggs in the contaminated ground; once ingested, the eggs hatch and develop into cysts in the internal organs. The most common mode of transmission to humans is by the accidental consumption of soil, water, or food that has been contaminated by the fecal matter of an infected dog. *Echinococcus* eggs that have been deposited in soil can stay viable for up to a year. | Chile | Lama and Vicugna/Camelidae. | Chile: Camelidae (1364) | Chile: 489 Camelidae import/875 transit | Persons with cystic echinococcosis often remain asymptomatic until hydatid cysts containing the larval parasites grow large enough to cause discomfort, pain, nausea, and vomiting. The cysts grow over the course of several years before reaching maturity and the rate at which symptoms appear typically depends on the location of the cyst. The cysts are mainly found in the liver and lungs but can also appear in the spleen, kidneys, heart, bone, and central nervous system, including the brain and eyes. Cyst rupture is most frequently caused by trauma and may cause mild to severe anaphylactic reactions, even death, as a result of the release of cystic fluid. | Risk from 1364 camelidae of which 489 are imported into the Netherlands. A canine is needed for transmission to humans. These camelidae probably live on farms, where are often also dogs living. Unlikely that the camelidae are fed to the dogs. Disease mild to severe, even death. | 2 | 2 | 1 | - |
| Japanese encephalitis virus | JE virus is transmitted to humans through the bite of infected *Culex* species mosquitoes, particularly *Culex tritaeniorhynchus*. The virus is maintained in a cycle between mosquitoes and vertebrate hosts, primarily pigs and wading birds. Humans are incidental or dead-end hosts, because they usually do not develop high enough concentrations of JE virus in their bloodstreams to infect feeding mosquitoes. | China, Indonesia, Japan, Philippines, Russia, Singapore, Vietnam | Wading birds, other bird species common to both Asia and North America, including English sparrows, house finches, pigeons, ducks and chickens, produce viraemia after experimental infection. However, these bird species do not contribute to epidemic JEV transmission to the same extent as ardeid birds and pigs, possibly because they only produce a low-level viraemia, and *Cx. Tritaeniorhynchus* rarely feeds on them. | Singapore: birds psittacidae (73) Sturnidae (12). Philippines: birds casuariidae (2) psittacidae (2) | Singapore 73 psittacidae transit 3rd countries/ 12 Sturnidae transit 3rd countries. Phillipines 2 casuariidae transit EU/2 psittacidae transit EU. | Among patients who develop encephalitis, 20% - 30% die. Although some symptoms improve after the acute illness, 30%-50% of survivors continue to have neurologic, cognitive, or psychiatric symptoms. | Risk from 89 birds, all-in transit. Especially wadings birds and pigs are a risk as it is not known if other bird species produce enough viraemia A vector is needed for transmission to humans. Disease is severe. | 2 | 2 | 2 | 2 |
| *Rickettsia africae*/ African tick bite fever | The disease is caused by *Rickettsia africae*, a recently identified spotted fever group rickettsia, which is transmitted by ungulate ticks of the Amblyomma genus in rural sub-Saharan Africa and the French West Indies. Humans are only accidental hosts. | South Africa, Kenya | Wild ungulates | South Africa: Giraffidae (53) Kenya:/ | Transit 3rd countries | African tick-bite fever is a bacterial infection that is spread through the bite of infected ticks. Symptoms usually appear within 2 weeks after a tick bite and often include fever, headache, muscle soreness, and a rash. At the site of the tick bite will be a red skin sore with a dark center. African tick-bite fever is typically milder than some other rickettsioses, but recovery is improved with treatment | Risk from 53 giraffidae, all in transit, probably animals from a zoo in transit to another zoo. Ticks needed for transmission, are the animals treated against ectoparasites? Disease is mild and easily treatable. | 1 | 1 | 1 | - |
| *Rickettsia typhii* | Humans become infected when they visit disease-endemic areas infested with rats and acquire infection by inhalation or by self-inoculating infected fleas or flea feces into skin. *Rickettsia typhi* is transmitted primarily by the rat flea, Xenopsylla cheopis, although lice and mites are also potential vectors. | Mexico, USA (Texas) | Commensal rodents (mainly *Rattus norvegicus,* and *Rattus rattus*) are considered the main reservoir, but other vertebrate hosts may serve as reservoir including house mice, shrews, opossums, skunks, and cats. | Mexico: Felidae (15). The specific species of rodents are not present in the database. | Transit 3rd countries | Murine typhus is a febrile illness caused by Rickettsia typhi. The clinical manifestations are nonspecific, and the signs and symptoms resemble those of several other febrile illnesses. Murine typhus can be a self-limiting infection; however, it should be diagnosed and treated because complications and even death can result | Risk from 15 felidae, they are all in transit. No rodents from Mexico or the USA. Transmission cycle not well understood yet, so role of other hosts unclear. A vector is needed for transmission to humans. These Felidae from Mexico are probably from a zoo and treated against ectoparasites. This disease can be lethal. | 1 | 1 | 1 | - |
| *Rickettsia rickettsii* | It is transmitted to humans by the bite of infected tick species. In the United States, these include the American dog tick (*Dermacentor variabilis*), Rocky Mountain wood tick (*Dermacentor andersoni*), and brown dog tick (*Rhipicephalus sanguineus)*. | North and South America | rodents and lagomorphs | Argentina: Rodents Dasyprocta (6) and Hydrochoerus(3) | Transit 3rd countries | Rocky Mountain spotted fever (RMSF) is a tickborne disease caused by the bacterium *Rickettsia rickettsii*. RMSF is a serious illness that can be fatal in the first eight days of symptoms if not treated correctly, even in previously healthy people. The progression of the disease varies greatly. | Risk from 9 rodents that were all-in transit. Transmission to humans by ticks. Disease can be fatal. | 1 | 1 | 1 | - |
| Rocio virus | The biological cycle of ROCV is not completely understood, but most likely involves an arthropod vector and susceptible vertebrate host (Fauquet et al. 2005). There is strong evidence that the virus circulates among ornithophilic mosquitoes and wild birds (Mitchell et al. 1986). | Brazil | Wild birds | Brazil: birds psittacidae (8) | Transit 3rd countries | Rocio virus (ROCV) has been responsible for epidemics of severe encephalitis in Brazil with a case-fatality rate of 10 % and development of sequelae in 20 % of the survivors. | Risk from 8 birds, all in transit. But transmission cycle not fully understood yet. Vector needed for transmission. Disease is severe. | 1 | 2 | 1 | 2 |
| Ross river virus | In Australia, the major vectors of Ross River virus to humans are various *Culex* and *Aedes* mosquitoes. No human to human transmission. | Indonesia | Marsupials (especially kangaroos and wallabies) are the most important vertebrate amplifying hosts | Indonesia: Diprotodontia (3) | Transit 3rd countries | Characterised by painful or swollen joints lasting from days to months. Symptoms usually settle by themselves. | Risk from 3 diprotodontia (and no kangaroos and wallabies) from Indonesia where not many cases of ross river virus are reported. All animals in transit. Vector needed for tranmission. Disease not lethal. | 1 | 1 | 2 | 2 |
| Saint Louis encephalitis virus | St. Louis encephalitis virus (SLEV) is maintained in a mosquito-bird-mosquito cycle, with periodic amplification by peridomestic birds and Culex species mosquitoes. It is transmitted to humans by the bite of an infected mosquito | Northern, Southern and Central America | Wild birds, domesticated fowl, rodents, and possibly other mammals. Primary reservoirs are wild birds, domestic fowl | Canada: birds Phoenicopteridae (7) Falconidae (3). USA: birds Ramphastidae (9) Phasianidae (7) Apterygidae (1). Argentina: Cariamidae (2) Spheniscidae (33). Brazil: birds psittacidae (8). Cuba: Cathartidae (10) Phoenicopteridae (480) Threskiornithidae (30). Peru: Alcedinidae (3) Anhimidae (5) Cathartidae (30) Falconidae (5) Momotidae (16) Psophiidae (4) Ramphastidae (12) Thraupidae (24) Trochilidae (15) Trogonidae (3). Suriname: Cotingidae (8) Psittacidae (1640), Ramphastidae (91) | Only 10 birds from USA import, all others transit 3rd countries or EU | Most persons infected with SLEV have no apparent illness. Initial symptoms of those who become ill include fever, headache, nausea, vomiting, and tiredness. Severe neuroinvasive disease (often involving encephalitis, an inflammation of the brain) occurs more commonly in older adults. In rare cases, long-term disability or death can result. There is no specific treatment for SLEV infection; care is based on symptoms | Risk from 2446 birds, only 10 birds import. Vector needed for pathogen transmission to humans. Most infected people have no symptoms. | 1 | 2 | 2 | 2 |
| *Salmonella* spp. | *Salmonella* can be transmitted from animals to humans directly and indirectly (via animal bedding, food, water etc.). | worldwide | While some serotypes are host-specific, all mammals, birds, reptiles and amphibians can become infected with *Salmonella* spp.. They can become subclinical carriers for months or even years (CFSPH, 2013). | Birds: total (2938)Amphibians: Anura (16985), Urodela (307). Reptiles: Crocodilia (1401), squamata (44593), testudines (422627) | Import: Birds: 121, Amphibians: 11979 Anura, 307 Urodela. Reptiles 957 crocodilia, 38936 squamata, 159614 testudines. Transit EU: Amphibians: Anura 4914, Reptiles: 44 crocodilia, 4901 squamata, 10514 testudines. Transit 3rd countries: Amphibians 92 Anura, Reptiles 400 crocodilia, 756 squamata, 252499 testudines. | Symptoms of persons infected with *Salmonella* spp. are predominantly diarrhoea, fever and abdominal cramps. Most people recover without treatment after 4 to 7 days, but some persons need to be hospitalized because of severe diarrhoea or extra-intestinal symptoms | There are more than 2500 different Salmonella serotypes, which differ in virulence. It is not possible to select the most virulent *Salmonella* spp., since the diversity of clinical symptoms depends on various factors (e.g. immunity, type of salmonella). However, the chance on exposure to (rare and exotic) *Salmonella* spp. is high since a large number of animals were imported that are probably destined for the pet industry. | - | 2/3 | 4 | - |
| Sindbis virus | Mosquitoes of genera Culex and Culiseta are considered the primary vectors to transmit SINV to humans, but the virus has been also isolated from Aedes and Anopheles mosquitoes. | Kenya, Uganda, Tanzania, South Africa, Egypt, Philippines | Birds might be involved in the natural cycle of SINV as important reservoirs, but spillover to other vertebrates may occur. Grouse (family: Phasianidae) and passerines (order passeriformes) are probable amplifying hosts for SINV. | No phasianidae from selected countries. Passeriformes from Tanzania: Corvidae (4) Nectariniidae (11) Ploceidae (11) Sturnidae (11). | All in transit to 3rd countries | The incubation period of SINV infection is often less than seven days, but has not been established. Maculopapular and often itchy exanthema over the trunk and limbs, mild fever, and joint symptoms, particularly in wrists, hips, knees, and ankles, are the hallmarks of acute SINV infection, sometimes accompanied by nausea, general malaise, headache, and muscle pain. Infectious and basic blood parameters are typically within normal range. In children, the clinical disease is usually mild, and can present without joint symptoms. Asymptomatic infections are not uncommon. Fatal infections have not been reported. | Risk from 37 birds, all-in transit. Vector needed for transmission. Disease is not lethal. | 1 | 1 | 2 | - |
| T-cell lymphotropic virus 1/HTLV-1 | The most important routes of HTLV-1 transmission were found to be from mother to child and predominantly through breastfeeding, sexual intercourse, and blood contact, including the transfusion of infected cellular products or sharing of needles and syringes. The transmission from STLV to HTLV is not fully understood. Consumers and hunters of bushmeat are at risk. | Japan, Africa, the Caribbean islands, and Central and South America emerging as the areas of highest prevalence in the world. Also in USA, but lower prevalence. | Primates (red colobus, sooty mangabeys etc.) | Peru: Aotidae (11) Cebidae (2) , USA: Galagidae (2), Ghana Cercopithecidae (1), Tanzania Cercopithecidae (75) | All transit to 3rd countries or EU | Two diseases have been definitely associated with HTLV-I: • adult T-cell leukaemia/lymphoma (ATLL). • HTLV-I associated myelopathy/tropical spastic paraparesis (HAM/TSP). Only a very small proportion of HTLV-I carriers will actually develop disease. | Risk from 91 primates, that were all-in transit. Only a small proportion of HTLV-1 carriers will develop disease. | 1 | 1 | 1 | 1 |
| Venezuelan equine encephalitis virus | Major outbreaks involving humans have been associated with Venezuelan equine encephalitis subtype I, varieties AB and C. All transmission of Venezuelan equine encephalitis is via mosquitoes. Not sure what the role of birds is in the transmission of VEEV | Colombia, Ecuador, Peru, Mexico, USA | Sylvatic rodents and wild birds | USA: birds apterygidae (1) ramphastidae (9) phasianidae (7). Peru: birds Alcedinidae (3) anhimidae (5) cathartidae (30) Falconidae (5) Momotidae (16) Psophiidae (4) Ramphastidae (12) Thraupidae (24) Trochilidae (15) Trogonidae (3) | USA: 1 apterygidae and 9 ramphastidae import/7 phasianidae in transit to EU. Peru: 5 Falconidae in transit to 3rd countries, all other birds from Peru in transit to the EU | Venezuelan equine encephalitis is an acute viral disease characterized by fever, chills, headache, nausea, vomiting, lumbosacral pain, and myalgia, which may progress to encephalitis. People with weakened or compromised immune systems, the young, and the elderly can become severely ill or die from this disease. | Risk from 134 birds, but not sure what the role of birds is in the transmission of VEEV. No rodents imported from countries with VEEV. Only 10 birds imported into the Netherlands. Vector needed for transmission. Disease: mild to severe. | 1 | 1/2 | 2 | - |
| West Nile virus | West Nile virus is most commonly transmitted to humans by mosquitoes. No transmission from person-to-person or from animal-to-person through casual contact. | Tanzania, USA, United Arab Emirates, South Africa, Argentina | Many bird species of different families are susceptible to WNV infection, but most birds are only viraemic for a few days Also found in mammals, reptiles and amphibians, but their role in the lifecycle is unknown. They are probably dead-end host. | Canada: birds phoenicopteridae: (7), falconidae (3). Tanzania: Ciconiidae (16), Threskiornithidae (9), Bucorvidae (10), Pelecanidae (10), Phoenicopteridae (60) USA: birds ramphastidae (9) phasianidae (7) Apterygidae (1) Brazil: birds psittacidae (8). South Africa: Ramphastidae (6). | Import: 9 ramphastidae and 1 Apterygidae from the USA, 16 Ciconiidae , 9 Threskiornithidae, 10 Bucorvidae, 10 Pelecanidae, 60 Phoenicopteridae from Tanzania. 6 Ramphastidae from South Africa. | In about 20% of the human cases symptoms develop, ranging from fever, headache, tiredness, body aches, nausea, vomiting, a skin rash (on the trunk of the body) and swollen lymph glands. Serious neurological problems occur in less than 1% of the human cases | Risk from 121 imported birds and 955 in transit. Low number of birds imported. Only a few bird species that develop a viraemia that is high enough to infect Dutch mosquitoes. Short viraemic period. Keep an eye on geographical spread of west nile virus. Transmission through mosquitoes. Import of mosquitoes is probably negligible as consignments of live animals from third countries have to be sprayed with insecticidal aerosols against vectors. Disease not severe in most people. | 2 | 2 | 2 | 2/3 |
| Western equine encephalitis virus | WEEV is normally maintained in wild bird populations, and Culex tarsalis appears to be the most important vector for this virus in North America | Western North America, including Canada, and as far south as Argentina | Passerine birds (order: passeriformes) are the usual reservoir hosts for WEEV, but this virus may also cycle in blacktail jackrabbit (Lepus califomicus) populations. Reptiles have been proposed as possible overwintering hosts. | Passerine birds from Suriname: Cotingidae (8) and from Peru: Thraupidae (24) Canada Reptiles (457), birds (10). USA reptiles (174260) birds (17). Argentina reptiles (250) birds (35). Chile / Ecuador /. Guyana, reptiles (2100) Peru reptiles (252565) birds (117) , Uruguay/,Colombia / Suriname reptiles (1901) birds (1739). | Passerine birds all in transit to EU. Reptiles Canada: 390 import/67 transit. Reptiles USA: 165252 import/9008 transit. Reptiles Argentina: 250 transit . Reptiles Guyana: 1595 import/505 transit. Reptiles Peru: 25 import/252540 transit. Reptiles Suriname: 639 import/1262 transit. | Causes asymptomatic or mild infections in humans, with non-specific symptoms such as sudden onset of fever, headache, nausea, vomiting, anorexia, and malaise. Some patients may also present with altered mental status and weakness, with signs of meningeal irritation. In rare cases, WEEV infection may cause encephalitis or encephalomyelitis, resulting in neck stiffness, confusion, visual disturbances, photophobia, tonic-clonic seizures, somnolence, coma, and death. | Risk from 1950 birds, all-in transit. If it's indeed in reptiles, the risk is a lot bigger as 167901 reptiles were imported and 263632 reptiles were in transit. A vector is needed for transmission. In most people asymptomatic or mild infections. | 2 | 2 | 2 | 2 |
| *Yersinia pestis* | Plague is a zoonotic disease affecting rodents and transmitted by fleas from rodents to other animals and to humans. Scientists think that plague bacteria circulate at low rates within populations of certain rodents without causing excessive rodent die-off. These infected animals and their fleas serve as long-term reservoirs for the bacteria. This is called the enzootic cycle. | Africa, Asia, South America. In the USA, Egypt and Argentina the presence of sylvatic foci of the plague are suspected (Dennis and Campbell, 2008; Lotfy, 2013). | > 200 species of rodents and lagomorphs have been implicated as reservoirs for *Yersinia pestis*, but the exact number of rodent species that are more than accidental reservoirs is uncertain (Dennis et al., 1999). Of the rodents in the database, Caviidae, muridae and dipodidae are linked to *yersinia pestis* in literature. | Argentina: Caviidae (3) Dasyproctidae (6). Egypt: Muridae (67) Dipodidae (150) | Egypt 67 Muridae and 150 Dipodidae import. Argentina 3 Caviidae and 6 Dasyproctidae transit 3rd countries. | Bubonic plague or [septicemic plague](http://www.cdc.gov/plague/symptoms/index.html). Bubonic plague is characterized by sudden onset of fever, headache, chills, and weakness and one or more swollen, tender and painful lymph nodes. When untreated, septicemic plague can develop. These patients develop bleedings in the skin and other organs which can lead to skin and tissues turning black and ultimately death of the patient. Pneumonic plague, the most serious form of the plague. These patients develop fever, headache, weakness, and a rapidly developing pneumonia with shortness of breath, chest pain, cough, and sometimes bloody or watery mucous. The pneumonia may cause respiratory failure and shock. Plague is a very serious illness, but is treatable with commonly available antibiotics. | Risk from 226 rodents of which 220 in literature linked to *Yersinia Pestis*. 217 import and 9 transit. Disease is severe but treatable. | 2/3 | 2/3 | 2 | - |

* Expert four scored only nine of the 18 pathogens as the other pathogens were outside her field of expertise
**The experts indicated that they did not have enough information to score this pathogen
